# Characterization of phyllosphere endophytic lactic acid bacteria reveals a potential novel route to enhance silage fermentation quality

**DOI:** 10.1038/s42003-024-05816-3

**Published:** 2024-01-22

**Authors:** Hongzhang Zhou, Shangang Jia, Yu Gao, Xiaomei Li, Yanli Lin, Fuyu Yang, Kuikui Ni

**Affiliations:** 1 College of Grassland Science and Technology, China Agricultural University, Beijing, 100193 China; 2 College of Animal Science, Guizhou University, Guiyang, 550025 China

**Keywords:** Applied microbiology, Microbiome

## Abstract

The naturally attached phyllosphere microbiota play a crucial role in plant-derived fermentation, but the structure and function of phyllosphere endophytes remain largely unidentified. Here, we reveal the diversity, specificity, and functionality of phyllosphere endophytes in alfalfa (*Medicago sativa* L.) through combining typical microbial culture, high-throughput sequencing, and genomic comparative analysis. In comparison to phyllosphere bacteria (PB), the fermentation of alfalfa solely with endophytes (EN) enhances the fermentation characteristics, primarily due to the dominance of specific lactic acid bacteria (LAB) such as *Lactiplantibacillus*, *Weissella*, and *Pediococcus*. The inoculant with selected endophytic LAB strains also enhances the fermentation quality compared to epiphytic LAB treatment. Especially, one key endophytic LAB named *Pediococcus pentosaceus* EN5 shows enrichment of genes related to the mannose phosphotransferase system (Man-PTS) and carbohydrate-metabolizing enzymes and higher utilization of carbohydrates. Representing phyllosphere, endophytic LAB shows great potential of promoting ensiling and provides a novel direction for developing microbial inoculant.

## Abstract (web summary)

The study reveals the diversity, specificity and functionality of phyllosphere endophytes in alfalfa silage fermentation and finds a unique microbe resource in phyllosphere endophytes which tends to enrich genes related to carbohydrate utilization.

## Introduction

The interactions between plants and microbial community play a vital role in maintaining ecosystems and promoting sustainable agricultural development^1,2^. Reportedly, phyllosphere is the largest biological surface on the earth, containing even more phyllosphere microorganisms than the quantity of plant cells^3^. Phyllosphere microbial communities include both epiphytes inhabiting on the leaf surface^4^ and endophytes living inside the plant^5^. With a high microbial diversity, they play a significant role in improving the productivity of agricultural systems as well as food safety and quality. For example, they could secrete unique substances to exert the host’s resistance to pathogens^6,7^, and promote nitrogen-carbon cycle with fixed nitrogen and methanol^4,8–10^. In addition, the colonization of undesirable phyllosphere microorganism also directly threatens the safety and quality of plant products^11,12^. Therefore, elucidating the relationship between plants, phyllosphere epiphytes and phyllosphere endophytes are significant to promote the sustainable production of crops and the safety and quality of agricultural products.

Nowadays silage is commonly used as the most important feed sources of nutrients for ruminants around the world. According to statistics, the annual demand for silage in China is estimated to be 737.09 million tons, markedly surpassing the present annual production rate of approximately 280 million tons^13^. By 2050, the world will require 60–70% more meat and milk than consumed today to feed an estimated 9.3 billion people on earth^14^, leading to a substantial escalation in future silage production. Alfalfa (*Medicago sativa* L.) is distributed in almost all temperate areas of the world and is a source of high-quality forage for ruminants, especially dairy cows, with the advantages of high protein content (16–24% dry matter) and good palatability. Ensiling has been considered as one of the most important ways for processing and preserving fresh alfalfa as animal feed, and complex microbial activities initiated by phyllosphere microorganisms are involved in fermentation, including synergy and competition of lactic acid bacteria (LAB), yeasts, molds, bacilli and enterobacteria. These microorganisms in fresh materials go through the whole ensiling process^15^, and the competitive microorganisms finally become the dominant community and shape the nutrient contents in alfalfa silage. Up to date, phyllosphere epiphytic bacteria have been considered mainly in silage production, and it is believed that the assembly of epiphytic microorganisms in alfalfa leaf is affected by non-biological factors, such as host, environment, temperature, moisture, radiation and so on^16,17^. However, our understanding of the diversity and function of phyllosphere endophytes in silage fermentation has lagged far behind. We hypothesized that phyllosphere endophytes, especially of endophytic LAB, were an important microbiome source that potentially determined the quality of forage silage fermentation^18,19^.

LAB are often detected inside the plant^20^, and play a certain role in promoting plant growth and inhibiting the propagation of pathogens^21^. Phyllosphere LAB are generally regarded as an important source of probiotics in fresh or fermented products, including silage, and play an increasingly important role in agricultural production and food health^22^. LAB could produce acids quickly and reduce pH value during silage fermentation, subsequently inhibiting harmful microbial activities and reducing nutrient loss^23,24^. Regarding alfalfa, it has been reported that low count of epiphytic LAB in alfalfa, together with low content of soluble carbohydrate and high buffer energy value, resulted in a poor quality of naturally fermented silage^25^. Thus, exogenous LAB are often used as inoculum to regulate the microbial communities and improve the silage quality. However, these exogenous sources of microorganisms usually hold a poor adaptability to the environment and an unstable inoculation effect. In contrast, endophytes inside the plants live in a stable environment with a tolerance to antibacterial substances produced by plants in the long-term coevolution process^26–29^. Many studies have shown that some endophytes, such as LAB, could metabolize and produce a variety of antibacterial or antioxidant substances including polyphenols, polysaccharides, flavonoids and so on^30,31^. Therefore, LAB inside alfalfa might achieve a strong environmental adaptability and gain the potential ability of improving fermentation quality.

The current study aimed to characterize phyllosphere endophytic microbiota inhabiting in alfalfa and its dynamics during ensiling process, and to use that information to understand whether phyllosphere endophyte could enhance silage fermentation quality. Phyllosphere epiphytes and endophytes resources were collected from the main alfalfa-producing areas in China. Then, conventional microbial culture, high-throughput sequencing and genomic comparative analysis were performed to elucidate the microbiota composition and function of phyllosphere endophytes. This approach helped to seek for the crucial endophytic microbiota associated with silage quality. Finally, the endophytic strains were inoculated to alfalfa silage to verify whether they could enhance silage fermentation quality. This study provides a new potential route for developing efficiently microbial inoculants using these phyllosphere endophytes.

## Results

### Diversity of culturable phyllosphere bacteria in fresh alfalfa

**Fig. 1:**
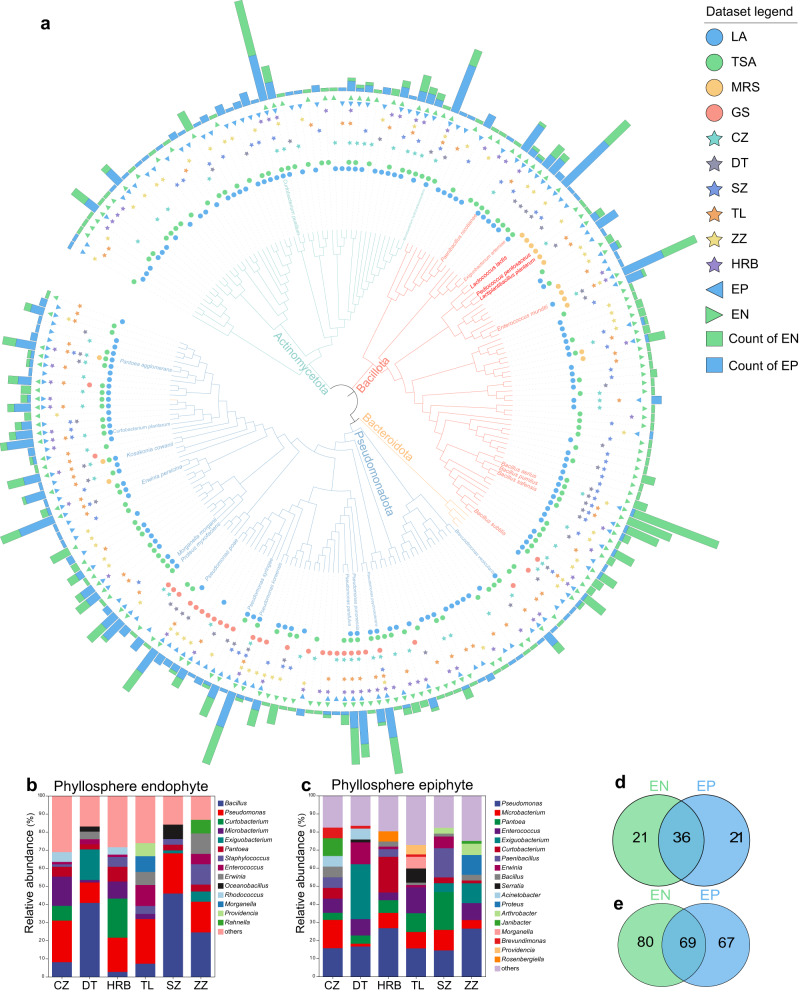
Phyllosphere bacteria in fresh alfalfa. **a** Phylogenetic tree based on the 795 culturable phyllosphere bacteria by 16S rRNA gene sequences. The line colors in the inner circle represent four phyla of Bacillota (red), Bacteroidota (orange), Pseudomonadota (blue), and Actinomycetota (green). The colors of outer circles indicate the four media culturable microorganisms were cultured on, i.e., LA LB nutrient agar, TSA soybean-casein digest agar medium, MRS Man Rogosa Sharpe agar medium, GS Gauze’s Synthetic Medium No.1. The stars colors represent the six regions, i.e., CZ Cangzhou, DT Datong, HRB Harbin, TL Tongliao, SZ Shuozhou, ZZ Zhuozhou. The triangle colors represent the endophytes (EN) or epiphytes (EP) isolated from alfalfa leaves, and the bars on the outer represent the microbial abundance. The lactic acid bacteria used as inoculants for the following alfalfa silage were highlighted in red, and the other phyllosphere bacteria with a high abundance were in other colors. Composition of endophytes (**b**) and epiphytes (**c**) were shown for six sampling regions. Venn plots were shown for endophytic and epiphytic bacteria in the levels of genus (**d**) and species (**e**).

To investigate the species and functions of epiphytes and endophytes in fresh alfalfa, a total of 795 strains from 58 healthy alfalfa samples were collected from six areas in China, based on typical microbial culture. A phyllosphere bacteria resource pool was established for fresh alfalfa, which consisted of 391 phyllosphere epiphytic and 404 phyllosphere endophytic strains. Through performing 16S rRNA gene sequencing analysis, we determined their taxonomies and assigned them to 216 species of 78 genera of 4 phyla (Fig. 1a, Supplementary Fig. 1, Supplementary Data 1). The three dominant phyla of Pseudomonadota, Bacillota, and Actinomycetota accounted for 46.54%, 32.08%, and 20.50% of the relative abundance of total bacteria, respectively, and the top three dominant phyla were identical for epiphytes and endophytes. A total of 149 species in 57 genera were identified as endophytes, and the dominant genera were *Bacillus* (20.79%), *Pseudomonas* (19.55%), *Curtobacterium* (5.45%), and *Microbacterium* (5.45%) (Fig. 1b). In contrast, with 136 species in 57 genera, the dominant genera in epiphytes included *Pseudomonas* (19.44%), *Microbacterium* (8.18%), *Pantoea* (7.93%), *Enterococcus* (7.67%), *Exiguobacterium* (7.67%) and *Curtobacterium* (5.12%) (Fig. 1c).

Epiphytes and endophytes showed a specificity among the six areas. For example, among endophytes, *Bacillus* was mainly found in Datong (DT), Shuozhou (SZ), and Zhuozhou (ZZ), *Curtobacterium* mainly in Harbin (HRB), and *Microbacterium* mainly in Cangzhou (CZ) (Supplementary Fig. 2a). Regional specificity was also observed in epiphytes, *Microbacterium* dominant in CZ, HRB, Tongliao (TL) and SZ, *Pantoea* in TL and SZ, and *Enterococcus* in TL, DT and ZZ (Supplementary Fig. 2b). It was discovered that *Pseudomonas* had no obvious regional difference in both endophytes and epiphytes.

Although significant bacterial differences were found between epiphytic and endophytic communities (Fig. 1d, e), 69 species of 36 genera were still shared, including *Pseudomonas, Bacillus, Microbacterium, Exiguobacterium*, and *Pantoea*. There were 67 specific epiphytic species of 21 genera, including *Clavibacter, Hafnia, Lelliottia* and *Moellerella*, while 80 endophytic species of 21 genera, including *Kocuria, Obesumbacterium* and *Pediococcus*.

### Dynamics of endophytes community during ensiling

**Fig. 2:**
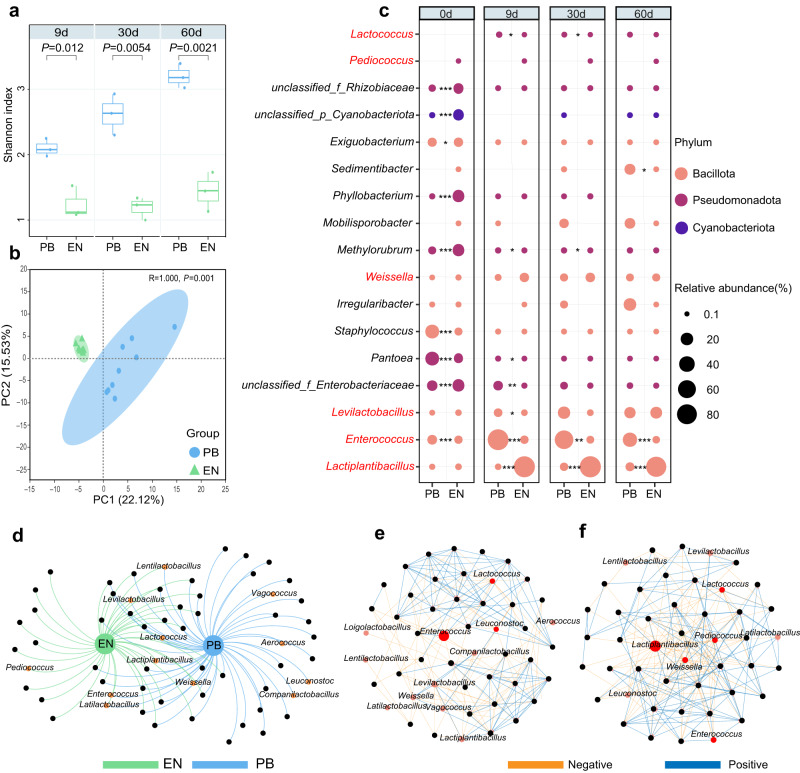
Microbial community of alfalfa silage initiated by endophytes. **a** The box-and-whiskers plot shows the Shannon index of silage microbial community in PB (silage fermentation by phyllosphere bacteria) and EN (endophytes) at three time points of 9, 30, and 60 days of fermentation. The box depicts the interquartile range (IQR) between the 25th and 75th percentiles respectively, the top of the box is the first quartile, the bottom is the third quartile, and the line within the box represents the median. The whiskers extend 1.5 times the IQR from the top and bottom of the box, respectively. Significant differences using two-tailed Welch’s *t*-test (*n* = 3, biologically independent samples). **b** PCA based on silage microbial community (*n* = 9, biologically independent samples). **c** Genus dynamics of silage microbial communities. The colors highlighted the phylum information. The circle size indicates the relative abundance, and the red font indicates lactic acid bacteria (LAB). Significant differences using two-tailed Welch’s *t*-test (*n* = 3, biologically independent samples). *P < 0.05; **P < 0.01; ***P < 0.001. **d** Co-occurrence network of microbial communities for the groups of PB and EN. LAB were highlighted in orange. Co-occurrence networks of microbial community were also shown for PB (**e**) and EN (**f**) respectively. LAB were highlighted in pink, and the key LAB was highlighted in red. The orange line represents negative correlation, and the blue line represents positive correlation (Spearman correlation > |0.6| and *P* < 0.05).

To study the dynamics of endophytic community during ensiling, sequencing analysis of 16S rRNA genes amplicon was performed. The Shannon-Wiener rarefaction curve showed that all treatment groups had a sufficient sequencing depth (Supplementary Fig. 3). The Shannon indexes on the 9th, 30th and 60th day showed an uptrend in phyllosphere bacteria treatment (PB), which were significantly higher than those of the endophyte group (EN) (*P* < 0.05) (Fig. 2a). It indicated a low diversity in the endophyte group during the process of fermentation. The principal component analysis (PCA) showed a significant difference in the beta diversity of bacterial communities between PB and EN groups (Fig. 2b). The fermentation in EN became relatively stable from the first sampling time point of 9 days, as the three time points of EN showed a large similarity (Supplementary Fig. 4a), while PB group had been experiencing significant microbiome shift as shown in PCA results (Supplementary Fig. 4b).

Before ensiling, fresh alfalfa had a complex microbiome structure, with significant differences between EN and PB (Fig. 2c). PB held a high abundance of *Staphylococcus, Pantoea, Enterococcus, unclassified_f_Enterobacteriaceae* and *Exiguobacterium*, while EN held a high abundance of *Phyllobacterium, Methylorubrum, unclassified_p_ Cyanobacteriota*, *unclassified_f_Enterobacteriaceae* and *unclassified_f_Rhizobiaceae*. With the prolonged ensiling time, *Pantoea* and *Staphylococcus* in the PB group were gradually substituted by *Enterococcus, Levilactobacillus* and *Lactiplantibacillus*. On the 60^th^ day, bacteria belonging to LAB as well as *Irregularibacter, Sedimentibacter, Mobilisporobacter* became the dominant genera. Compared with PB group, the EN group were dominated by *Lactiplantibacillus, Weissella* and *Levilactobacillus*. During the whole ensiling period, *Lactiplantibacillus*, *Weissella* and *Pediococcus* were more abundant in EN than those in PB, while more *Enterococcus* were found in PB. We compared the two groups for specific microorganisms and found LAB present in both groups during fermentation including *Lactiplantibacillus, Enterococcus, Levilactobacillus, Weissella, Lentilactobacillus, Lactococcus* and *Latilactobacillus*, while *Pediococcus* was found only in EN, and *Vagococcus, Aerococcus, Leuconostoc* and *Companilactobacillus* only in PB (Fig. 2d). The microbial co-occurrence network analysis also showed a different structure for EN and PB (Fig. 2e, f). *Lactococcus*, *Enterococcus* and *Leuconostoc* were key LAB genera of the PB group, with a node related degree beyond 10, while key LAB genera of the EN group were *Lactiplantibacillus, Weissella, Lactococcus, Leuconostoc* and *Pediococcus*. In summary, great differences were observed between endophytes fermentation and natural fermentation with phyllosphere bacteria.

### Effects of endophytes on silage fermentation

We further investigated the chemical characteristics of alfalfa silage (Supplementary Table 1). In terms of chemical properties, the EN group had a significantly higher contents of dry matter (DM), water-soluble carbohydrates (WSC), and crude protein (CP) (*P* < 0.05), as well as a significantly lower contents of ether extract (EE) (*P* < 0.05). The fermentation characteristics showed a significantly lower contents of ammoniacal nitrogen (NH_3_-N), acetic acid (AA), and pH, as well as a significantly higher contents of lactic acid (LA) (*P* < 0.05) in EN treatment (*P* < 0.05). Furthermore, the EN group had a significantly lower amount of enterobacterium and yeasts (*P* < 0.05).

**Fig. 3:**
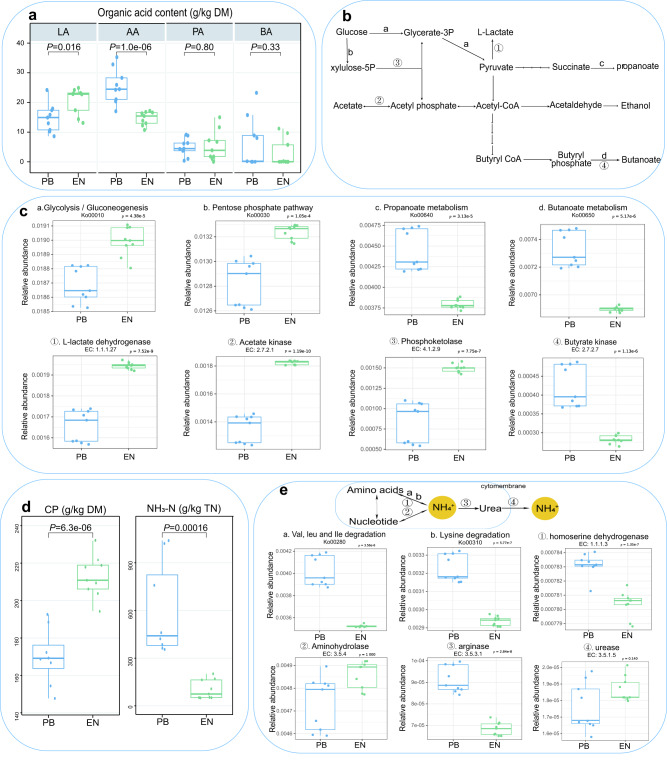
Fermentation quality of alfalfa silage caused by endophytic. **a** Organic acid content of alfalfa silage. **b** Schematic diagram of microbial organic acid metabolic pathway on the basis of KEGG. **c** KEGG enzyme activity based on Tax4Fun. **d** Crude protein content of alfalfa silage. **e** Schematic diagram of microbial deamination metabolic pathway and the KEGG enzyme activity was organized on the basis of KEGG. The box-and-whiskers plot shows the organic acid content, crude protein content and the relative abundance of each KEGG enzyme activity. The box depicts the interquartile range (IQR) between the 25th and 75th percentiles respectively, the top of the box is the first quartile, the bottom is the third quartile, and the line within the box represents the median. The whiskers extend 1.5 times the IQR from the top and bottom of the box, respectively. Significant differences using two-tailed Welch’s *t*-test (*n* = 9, biologically independent samples). LA lactic acid, AA acetic acid, PA propionic acid, BA butyric acid, CP crude protein, NH_3_-N Ammonia nitrogen/TN (total nitrogen), PB naturally silage by phyllosphere bacteria, EN endophyte.

The organic acid metabolism and amino acid degradation activities of microbial communities are closely related to the accumulation of organic acids and nutrient preservation during silage fermentation process. During silage fermentation process, the higher lactic acid content in the EN group was attributed to the significantly higher abundance of the glycolysis, the pentose phosphate pathway, and L-lactate dehydrogenase (*P* < 0.05). Meanwhile, acetate kinase activated acetic acid and involved it into energy and carbon metabolism, resulting in a lower acetic acid content. And the significantly (*P* < 0.05) lower abundance of butanoate metabolic pathway and butyrate kinase also resulted in a lower butyric acid content (mean ± S.E.M. = 2.97 ± 1.55 g/kg DM) than that in PB group (mean ± S.E.M. = 6.23 ± 2.84 g/kg DM), although not statistically significant (*P* = 0.33) (Fig. 3a–c, Supplementary Table 1). During the deamination process of amino acids, we found that the EN group was significantly inferior to the PB group in the valine, leucine and isoleucine degradation, the lysine degradation, and the relative abundance of homoserine dehydrogenase, arginase (Fig. 3d, e). As a result, the EN group had a lower concentration of extracellular ammonia nitrogen and maintained the relatively high crude protein content in alfalfa silage.

### Selected endophytic LAB and their roles in alfalfa silage

**Fig. 4:**
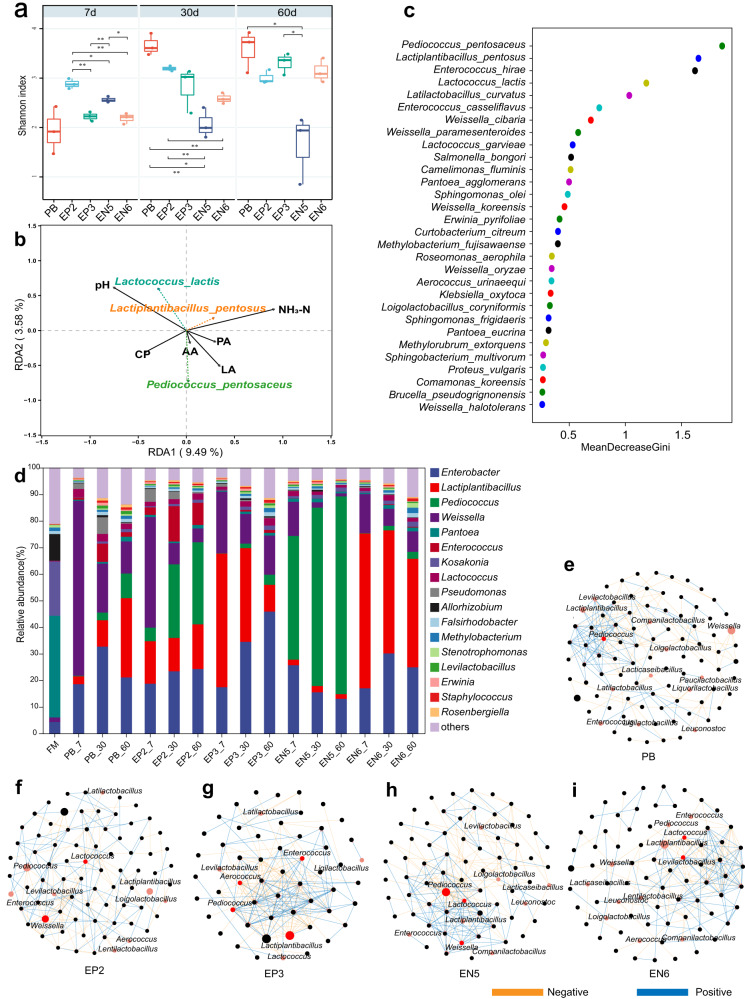
Microbial community of alfalfa silage with endophytic lactic acid bacteria (LAB) as additive. **a** The box-and-whiskers plot shows the Shannon index of silage microbial community. The box depicts the interquartile range (IQR) between the 25th and 75th percentiles respectively, the top of the box is the first quartile, the bottom is the third quartile, and the line within the box represents the median. The whiskers extend 1.5 times the IQR from the top and bottom of the box, respectively. Statistical significance (*P* < 0.05) among the treatments according to one-way ANOVA and LSD test at the 5% level (*n* = 3, biologically independent samples). *P < 0.05; **P < 0.01. **b** Redundancy analysis for microbial community and silage quality. **c** Random Forest analysis (decision tree = 500) of characteristic microorganisms of different silage microbial communities. **d** Silage microbial community composition in genus level. **e**–**i** Co-occurrence network of silage microbial community of PB (**e**), EP2 (**f**), EP3 (**g**), EN5 (**h**), EN6 (**i**). Pink node represents LAB, and red node represents key LAB. The orange line represents negative correlation, and the blue line represents positive correlation (Spearman correlation > | 0.6 | and *P* < 0.05). FM fresh matter of alfalfa, PB naturally silage by phyllosphere bacteria, EP2 inoculated epiphytic LAB *Lactococcus lactis,* EP3 inoculated epiphytic LAB *Lactiplantibacillus pentosus,* EN5 inoculated endophytic LAB *Pediococcus pentosaceus,* EN6 inoculated endophytic LAB *Lactiplantibacillus plantarum*.

LAB were the dominant organisms during silage fermentation, and we found specific LAB in endophytic communities. To confirm the regulatory effect of endophytic LAB on alfalfa fermentation, we selected eight LAB strains isolated from alfalfa. Then, the eight LAB strains (Supplementary Table 2) were used to prepare silage additives and added to fresh alfalfa (Supplementary Table 3) for determining fermentation indexes. However, of which only four treatments showed enhanced fermentation quality such as increased lactic acid content compared with PB group. Therefore, only these four treatments were presented and discussed here. Additionally, 16S rRNA genes in silage samples of PB, EP2, EP3, EN5 and EN6 groups were further sequenced based on PacBio Sequel II platform. The Shannon-Wiener rarefaction curve showed a sufficient sequencing depth for each sample (Supplementary Fig. 5). What was interesting is that except the EN5 treatment group with a downtrend of Shannon index during fermentation, PB, EP3, and EN6 showed an uptrend of Shannon index after silage fermentation and EP2 maintained a stable trend (Fig. 4a). This suggested EN5 owned a lower diversity of bacterial communities than the other treatments. PCA result showed a significant difference in the beta diversity of bacterial communities between the EN5 group and other groups (Supplementary Fig. 6).

We performed the redundancy analysis to measure the contribution of microbial communities to fermentation quality of alfalfa silage. Figure 4b showed that *Pediococcus pentosaceus* was positively correlated with contents of LA, AA, CP, but negatively correlated with pH and NH_3_-N. *Lactiplantibacillus pentosus* also showed positive and negative correlations with LA and pH, respectively, but was negatively and positively correlated with CP and NH_3_-N, respectively. Random forest analysis further confirmed that *P. pentosaceus* and *L. pentosus* were the most characteristic microorganisms in these treatment groups (Fig. 4c).

Before ensiling, bacterial communities in fresh matter of alfalfa (FM) were dominated by *Pantoea, Kosakonia, Allorhizobium*, and *Enterobacter* which were with a high relative abundance (Fig. 4d). With the extension of ensiling, LAB became dominant species, and the most dominant genera *Pantoea* was replaced by *Lactiplantibacillus* in PB group. The EP2 (*Lactococcus lactis*) group also experienced the same dynamic changes of microbiome like PB, but in EP2_60, *Pediococcus* became the most abundant, while in the EP3 (*L. pentosus*) group, *Enterobacter* became the most abundant in EP3_60, rather than *Lactiplantibacillus*. However, both EN5 and EN6 groups did not show significant shifts of microbiome during fermentation, unlike the two groups of EP2 and EP3. Specifically, EN5 (*P. pentosaceus*) and EN6 (*Lactiplantibacillus plantarum*) were dominated by *Pediococcus* and *Lactiplantibacillus*, respectively, which indicated their strong roles in the microbial assembly during ensiling process. Our endophytic LAB inoculum imposed a significant effect on the competition of microbiome in alfalfa silage. Therefore, we believed that *P. pentosaceus* EN5 and *L. pentosus* EN6 were the key microorganisms that caused the differences in microbial communities and fermentation quality among different treatment samples.

We built bacterial co-occurrence network for these five groups of PB, EP2, EP3, EN5, and EN6 (Fig. 4e–i), and found the different key LAB modules, for example, the key LAB genera of *Pediococcus* in PB group, *Weissella* and *Lactococcus* in EP2 group, *Pediococcus, Lactiplantibacillus, Aerococcus* and *Enterococcus* in EP3 group, *Pediococcus, Lactococcus* and *Weissella* in EN5 group, and *Levilactobacillus* and *Lactococcus* in EN6 group. As key LAB, *Pediococcus* had a negative correlation with most microorganisms only in EN5 group (see orange lines in Fig. 4h), but with many positive correlations in PB and EP3 (see blue lines in Fig. 4e, g). In summary, the results showed that the endophytic LAB, such as *P. pentosaceus* showed excellent traits for improving alfalfa fermentation in our selected candidate strains.

### Carbohydrate utilization by endophytic LAB in alfalfa silage

**Fig. 5:**
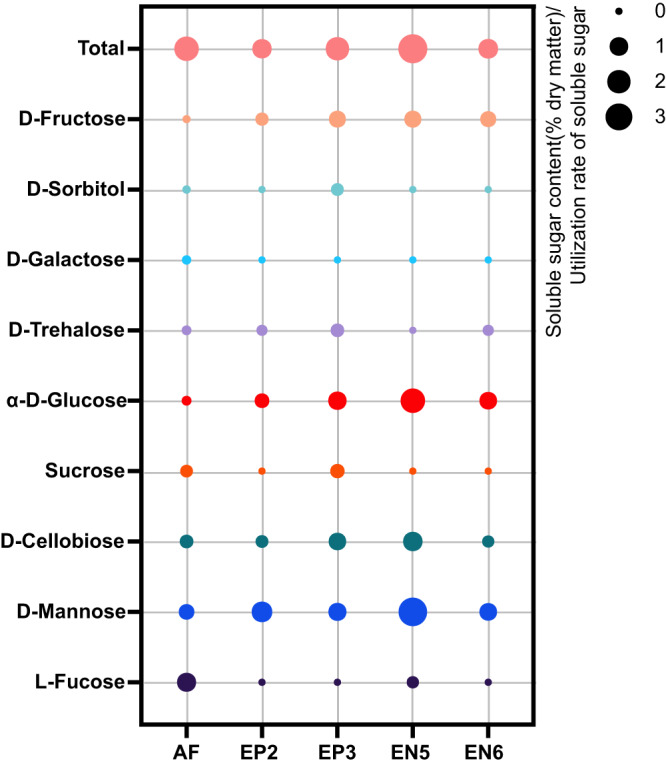
Utilization rate of soluble sugar in alfalfa by phyllosphere lactic acid bacteria (LAB). AF soluble sugar content of alfalfa (% dry matter), EP2 epiphytic LAB *Lactococcus lactis,* EP3 epiphytic LAB *Lactiplantibacillus pentosus,* EN5 endophytic LAB *Pediococcus pentosaceus,* EN6 endophytic LAB *Lactiplantibacillus plantarum*.

The growth and metabolism capacity of microorganisms is closely correlated with their growing environment. LAB produce large amounts of lactic acid using carbohydrates as substrates. Their acid production rate is closely correlated with the content and types of carbohydrates in the environment. We continue to study the biochemical characteristics of EP2, EP3, EN5 and EN6. Under laboratory conditions in the early stage, EN5 was inferior to EP3 and EN6 in the growth and acid production capacity in MRS broth (Supplementary Fig. 7), but superior to other strains in the performance in alfalfa silage. We inferred that endophytes living inside alfalfa are characterized by a high specificity in the utilization of tissue nutrients of alfalfa, especially the sugars of alfalfa. Therefore, we measured the soluble carbohydrate composition in fresh alfalfa and the utilization rate of different sugars by endophytes (Fig. 5). The results showed that soluble sugars in fresh alfalfa were mainly composed of L-fucose, D-mannose, D-cellobiose, sucrose, and α-D-glucose. Among the four strains of LAB selected, endophytic LAB of EN5 showed the highest comprehensive utilization rate of soluble sugars in alfalfa fresh materials, and strong absorption and utilization capacities of D-mannose, α-D-glucose, and D-cellobiose.

### Genome analysis of four phyllosphere LAB

**Fig. 6:**
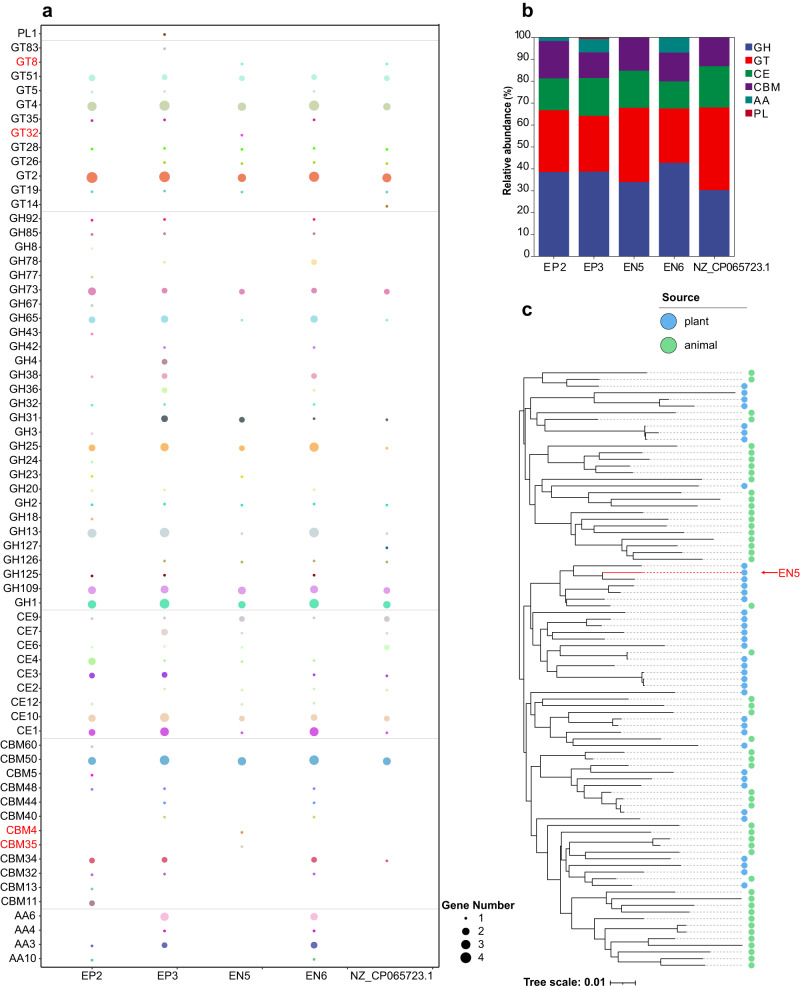
Comparative genomic analysis on genome assemblies of four selected lactic acid bacteria in alfalfa. **a** individual CAZymes for the degradation potential of polysaccharides by five genome assemblies, including the four ones in this study and the publicly available one of *Pediococcus pentosaceus* (accession of NZ_CP065723.1 in NCBI). The CAZymes unique in EN5 are marked in red. **b** The relative abundance of six CAZymes modules encoded in five lactic acid bacteria. **c** Phylogenetic trees based on the downloaded genome assemblies of *P. pentosaceus* from NCBI. The phylogenetic tree was constructed using the neighbor-joining method. Plant and animal sources are indicated by blue and green circles respectively.

We sequenced and assembled the four selected phyllosphere LAB, i.e., EP2, EP3, EN5, and EN6, the general genomic features among the four LAB species are listed in Table 1. Their respective genome sizes were 2,348,459 bp (*L. lactis* EP2), 3,582,264 bp (*L. pentosus* EP3), 1,843,636 bp (*P. pentosaceus* EN5) and 3,290,693 bp (*L. plantarum* EN6). We found many transport protein genes in these four strains of LAB, including MFS gene, ABC gene and PTS gene related to carbohydrate transport. Due to the restriction of genome size, the number of various transport protein genes of EN5 is relatively small except for PTS gene. We conducted a comparative analysis with another genome assembly of *P. pentosaceus* (NCBI accession of NZ_CP065723.1), and focused on the carbohydrate transport and metabolism capacities of LAB. It showed that in terms of the sugar transport capacity of LAB, in the mannose phosphotransferase system, EN5 contains two IIB genes, which have not been found in other four LAB. In addition, two IID genes were found in EN5, but only one existed in EP2, EP3 and EN6 (Supplementary Table 4). Based on the annotation results against carbohydrate-active enzymes (CAZymes) database (Supplementary Data 2), the five LAB species contained six families of CAZymes, including polysaccharide lyase (PL), glycosyl transferase (GT), glycoside hydrolase (GH), carbohydrate esterase (CE), carbohydrate-binding module (CBM) and auxiliary activities (AA). Except that the most abundant CAZymes gene in *P. pentosaceus* EN5 and *P. pentosaceus* NZ_CP065723.1 belongs to GT family, other LAB species CAZymes belong to GH family, and only *L. pentosus* EP3 contained the PL gene, AA gene were absent in *P. pentosaceus* EN5 and *P. pentosaceus* NZ_CP065723.1. EN5 had specific active enzyme correlated with glucose and mannose utilization, namely CBM35, CBM4, GT32, and GT8, compared with epiphytic strains of EP2 and EP3. Meanwhile, compared with the strain NZ_CP065722.3, there were three specific active CAZymes in EN5, namely CBM35, CBM4 and GT32 (Fig. 6a, b).

**Table 1:** Features of genome assemblies of four selected phyllosphere lactic acid bacteria isolated from alfalfa.

	EP2	EP3	EN5	EN6
Species	*Lactococcus lactis*	*Lactiplantibacillus pentosus*	*Pediococcus pentosaceus*	*Lactiplantibacillus plantarum*
Genome size (bp)	2,348,459	3,582,264	1,843,636	3,290,693
Contig N50 (bp)	2,348,459	3,556,640	1,802,413	3,290,693
Plasmid (bp)	None	25,624	41,223	None
Genes Number	2211	3172	1785	3092
Total length (bp)	2,023,869	2,868,987	1,622,142	2,760,264
Average gene length (bp)	915	904	908	892
Max gene length (bp)	6528	11,973	6021	8697
Min gene length (bp)	93	90	90	90
Repetitive sequence content (%)	0.29	0.12	0.2	0.11
GC content (%)	35.03	46.37	37.35	44.44
MFS gene number	23	56	18	43
ABC gene number	142	201	81	180
PTS gene number	19	62	21	55
Transport protein number	580	823	459	778

*EP2* epiphytic *L. lactis,* *EP3* epiphytic *L. pentosus,* *EN5* endophytic *P. pentosaceus,* *EN6* endophytic *L. plantarum*. *MFS* major facilitator superfamily, *ABC* ATP-binding cassette superfamily, *PTS* phosphotransferase system.

Due to the performance of EN5, we downloaded all the genome assemblies of *P. pentosaceus* in NCBI GenBank and carried out a comparative genomic analysis (Supplementary Table 5). The numbers of core genes were 998. The core-genome size gradually decreased with the sequential addition of genomes of newly sequenced isolates and became stable when the number of genomes was around 65. The pan-genome of the 90*P. pentosaceus* isolates comprised 5630 gene families (Supplementary Fig. 8).

The results of functional analysis of the core-, accessory- and unique- genome assigned to specific KEGG functional categories are shown in Supplementary Fig. 9. The major KEGG functional categories of core-genome were carbohydrate metabolism; translation; overview; nucleotide metabolism; replication and repair; membrane transport; energy metabolism, and amino acid transport and metabolism. The accessory genes mainly fell into the categories of carbohydrate metabolism; membrane transport; overview; amino acid metabolism; replication and repair; nucleotide metabolism; lipid metabolism, and metabolism of cofactors and vitamins. The unique genes mainly fell into the categories of carbohydrate metabolism; membrane transport; amino acid metabolism; replication and repair; overview; nucleotide metabolism; metabolism of terpenoids and polyketides, and glycan biosynthesis and metabolism. Compared to the core-genome, the KEGG categories of carbohydrate metabolism; membrane transport, and amino acid metabolism were enriched in the accessory- and unique- genome in these three cases. The phylogenetic tree was built based on the pan-genome profile and showed that the *P. pentosaceus* strains in the branches of the tree could be attributed to two habitats, including plant and animal (Fig. 6c). Notably, EN5 contained 9 unique proteins including SecB, which are not found in the other genome assemblies of *P. pentosaceus* (Supplementary Table 6).

## Discussion

Silage fermentation is a complex microbial activity, involving synergy and competition of a variety of microorganisms in silage fresh materials during the whole process of silage fermentation^15^. It has been widely considered that epiphytes naturally attached to fresh forages are the main microorganisms for initiating silage fermentation^19,32^. However, up to date, few studies were performed on discovering endophytes in alfalfa and other forages, and evaluating their effects on silage fermentation.

Microbial communities have various regulatory effects on silage fermentation^33^. From the perspective of the quantity and composition of microorganisms, we have found that there are differences in the composition of phyllosphere microorganisms of alfalfa in different regions, and the current view is that the lactic acid production ability and quantity of LAB in alfalfa raw materials is the main reasons for the fermentation quality of alfalfa silage^19,25^, and yet there are few reports on the impact of different types of microorganisms other than LAB on alfalfa fermentation, such as many aerobic bacteria. In this study, the differences in the composition and function of raw microorganisms between the phyllosphere bacteria group and the endophyte group were important factors for distinct silage fermentation qualities. We detected almost 10^4^ times as many aerobic bacteria as LAB in phyllosphere microorganisms of fresh alfalfa materials, and confirmed the large counts of *Staphylococcus, Pantoea, Pseudomonas, Enterobacter, Enterococcus, Microbacterium, Exiguobacterium* and *Curtobacterium* on the surface of fresh alfalfa forage using single-molecule real-time (SMRT) sequencing technology and microbial culture, which were supported by the previous results^34–38^. Phyllosphere bacteria in fresh alfalfa competed with LAB and consumed large amounts of nutrients in the early stage of silage fermentation. After 9 days of fermentation, *Enterococcus* and *Enterobacteriaceae* became dominant microorganisms in the natural fermentation group. *Enterococcus* is a kind of LAB with a weak acid production capacity, and could exist in large quantities in silage but cannot improve the silage fermentation quality^39^. The mass propagation of *Enterococcus* resulted in a high pH value and a low lactic acid content in the natural fermentation group on the 9^th^ day. This environment was not conducive to inhibiting the growth of *Enterobacteriaceae* and other harmful microorganisms. With the progress of fermentation, the α diversity of bacterial communities increased continuously, and some LAB with a strong fermentation capacity and lactic acid-producing bacteria began reproducing. Among endophytic communities in fresh alfalfa, as *Pantoea, Staphylococcus* and other aerobic microorganisms decreased significantly, their endophytic LAB reproduced quickly to become key microorganisms and produced a large amount of lactic acid to lower the pH of silage, subsequently inhibiting the growth of undesirable microorganisms and reducing the nutritional loss of silage.

The prediction of bacterial metabolism of organic acid and amino acid deamination during ensiling also confirmed that the differences in bacterial metabolism and function led to distinct silage fermentation qualities. Pyruvic acid was the main carbon skeleton in the process of organic acid production, and was metabolized and generated through glycolysis or pentose phosphate pathway. Our results indicated that endophytic communities had a higher glycolysis and pentose phosphate metabolic activity, and more abundant organic acid metabolic substrates. L-lactate dehydrogenase was the key enzyme in the conversion of pyruvic acid to lactic acid. The high lactic acid content in endophytic communities was attributed to the large count of LAB in the communities and their L-lactate dehydrogenase activity. Propionic acid was usually converted gradually from pyruvic acid through oxaloacetic acid and succinic acid, and could also be converted from lactic acid through acrylic acid^40,41^. The abundance of propionate metabolic pathway in endophytic communities was predicted to be lower than that in natural silage probably due to its simple community composition and weak propionic acid metabolism of LAB^42^. Butyric acid was converted gradually from pyruvic acid through acetyl CoA and butyric CoA^43^. The low butyric acid content in endophytic communities was attributed to the low count of butyric acid-producing bacteria in the communities and the low butanoate metabolism and butyrate kinase activity. Acetic acid was a common product of bacterial sugar metabolism and could also be involved in the synthesis of various metabolites as a substrate. We predicted a higher abundance of acetate kinase activity in endophytic communities, suggesting more active acetic acid metabolism in endophytic communities. Meanwhile, its low acetic acid content indicated that acetic acid in endophytic communities was more inclined to synthesis of other substances, such as butyric acid^44^. Reducing the NH_4_^+^ formation and emission was of great significance for the preservation of silage. NH_4_^+^ in silage was mainly sourced from the deamination of amino acids and nucleotides. We predicted a lower amino acid deamination pathway and enzyme activity in endophytic communities. Meanwhile, the activity of arginase, a key enzyme in the urea cycle^45^, was also obviously lower than that in natural silage. According to the findings of Hao et al. ^46^, protein-degrading bacteria in silage mainly included *Curtobacterium, Bacillus, Paenibacillus*, *Enterococcus, Pediococcus* and *Lactiplantibacillus*, with the highest proteinase activity in *Bacillus, Enterococcus*, *Clostridium* and *Enterobacter*. According to the results, free amino acids produced by microorganisms through protein hydrolysis should be balanced with their amino acid and nucleotide metabolism as available nitrogen sources. In the natural fermentation group, *Enterococcus, Clostridium* and *Enterobacter* with a high abundance decomposed protein in large quantities but couldn’t utilize it effectively, resulting in efflux of a large amount of NH_4_^+^ and then significant protein loss and degradation of silage quality.

This study found that endophytic *P. pentosaceus* EN5 in alfalfa had better fermentation characteristics during silage fermentation. In the early stage of experiment, *Pediococcus* was found to exist specifically in endophytic communities of fresh alfalfa and silage of the endophytic community group and considered as the key LAB in the endophytic fermentation treatment. *Pediococcus* is a type of LAB that widely exist in animals, fermented food, aquatic products, and plant products, and has attracted more and more attention as a potential probiotic object in recent years owe to its antimicrobial abilities, anti-inflammation, anticancer, antioxidant, detoxification, and lipid-lowering abilities^47^. *Pediococcus* was believed to be a good inoculant for silage^48–50^. However, *P. pentosaceus* was usually isolated from fermented silage, without any studies on the surface of or inside its fresh samples. It is worth noting that unlike epiphytes *L. lactis* EP2 and *L. pentosus* EP3, endophytes *P. pentosaceus* EN5*, L. plantarum* EN6 were added to alfalfa fresh materials as inoculants, then their bacterial communities could maintain a long-term stable state. Previous study results^48^ showed that no matter whether *P. pentosaceus* was added, *Lactiplantibacillus* was the dominant bacterial genus, which was inconsistent with this test. Random forest and redundancy analysis were used to confirm that *P. pentosaceus* EN5 had a better regulatory effect on alfalfa fermentation than other strains.

The effect of different phyllosphere microorganisms on the bacterial networks in alfalfa silage was investigated using the bacterial correlation network. Our results indicated that *Pediococcus* could be negatively correlated with more microorganisms in most cases, and more likely to become central species than other LAB. According to the previous report^51^, the negative interaction may weaken the competitive relationship of bacterial communities, while more negative interactions suggested a weaker competition among microbial communities and a more stable microbial network structure. In this study, the higher edge count of negative correlation with LAB suggested a more stable but less complex bacterial network structure. Besides, the edge count of negative correlation with LAB after the silage fermentation added with inoculants was higher than that without LAB inoculants, indicating a more stable but less complex bacterial network structure, which corresponded to the α diversity index of communities.

*Pediococcus pentosaceus* EN5 as a silage inoculant showed better adaption to the silage environment. For one thing, *P. pentosaceus* was always the key microorganism in bacterial communities of silage after inoculation with EN5, which was not found in other epiphytic strains in this experiment. Inoculation with EN5 made the pH of silage drop faster to the lowest level, which was also inconsistent with our previous evaluation of its growth curve and acid production rate in MRS broth. EN5 was inferior to EP3 and EN6 in the growth rate and the acid production rate in MRS Broth. It was generally believed that LAB took monosaccharides and oligosaccharides in plants as substrates for energy metabolism and production of lactic acid, acetic acid, ethanol and other small molecules of acids and alcohols. Nevertheless, various strains had distinct utilization rates of different sugars, which may be an important factor affecting the effect of silage inoculants. Endophytes, which existed in the internal environment of plants, were more advantageous over other microorganisms in the long-term coevolution process in terms of host nutrient utilization^52,53^. On that basis, we inferred that the higher utilization rate of host nutrients of EN5 was the main factor for its excellent performance in silage regulation. After the determination of the sugar components in alfalfa fresh materials and the sugar utilization capacity of corresponding sugars of strains, the results showed that EN5 surpassed other strains in the utilization capacity of main sugars in alfalfa, with the highest comprehensive utilization capacity to total alfalfa sugars. In the fresh alfalfa phyllosphere, the low amount of *Pediococcus* cannot overcome the survival competition^54,55^ and dispersal limitation^56^ caused by a large number of external microorganisms, so *Pediococcus* cannot dominated the fermentation, but after inoculation with EN5 silage additive, *Pediococcus* has the advantage of quantity and overcomes the source or metabolism limitation^12^ of species dispersal and rapidly dominates the fermentation of alfalfa silage by its unique adaptability to alfalfa sugar sources and strong adaptability. Nevertheless, *P. pentosaceus* has been reported to cause clinical infections while it is being promoted as an effective inoculant in fermented foods. Antibiotic resistance (AR) genes in this species are a matter of concern for treating clinical infections^57,58^. In this study, we found an AR gene associated with penam and cephalosporin in EN5 strain (Supplementary Table 7). Therefore, further research is needed on the resistance of EN5 to antibiotics. It should be verified, whether the rapid dispersal and domination of EN5 can cause the transfer of AR to pathogens in the environment and have an impact on the environment and animal health.

Additionally, LAB could grow in environments rich in a variety of sugar sources, which owed to its rich sugar transport systems and sugar-metabolizing enzymes^59^. Transmembrane transport of nutrients in organisms depends mainly on protein transporters in cell membranes. Phosphoenolpyruvate (PEP)-phosphotransferase (PTS) system was widely found in bacteria, fungi, and some archaea. Various sugars and their derivatives were phosphorylated by phosphorylation cascade and then transported into cells^60^. Unlike the other three strains, endophyte EN5 was found to have more genes encoding enzyme II complex of the PTS Mannose-Fructose-Sorbose (Man) Family in its genome. The PTS Man Family mediated the transport and phosphorylation of mannose, fructose and sorbose^61^, which may be one of the factors for the rapid utilization of alfalfa sugars by EN5. In addition to PTS, primary and secondary transport protein families on biological membranes were classified into two categories: ABC superfamily (ATP-binding cassette superfamily) and MFS (major facilitator superfamily). They occupied almost all transport proteins encoded in genomes of microorganisms or organisms^62,63^. We found large amounts of ABC and MFS transporters in the four strains. The transporters were predicted to be involved in the transport of carbon sources, but their substrate specificity for carbon absorption could not be predicted unfortunately. Once sugars were transported into cells, they were used as a carbon source for growth and produced energy through fermentation. Complete glycolysis and pentose phosphate pathway enzyme genes were found in genomes of the five LAB strains, suggesting that sugars could produce energy through fermentation by EMP or pentose phosphate pathway. As expected, none of the genomes of the five LAB encoded complete tricarboxylic acid cycle enzymes^64^. Restricted by the genomes of their strains, different LAB were programmed to make use of many different carbon sources specifically and efficiently. Xylan, mannan and xyloglucan were key polysaccharides in plant cells. Widely distributed in the structures and storage tissues of plants, they are potential carbon sources for microbial growth. These polysaccharides could not be hydrolyzed without the synergy of various carbohydrate enzymes. The CAZymes database described relevant catalytic and carbohydrate-binding module families (or functional domains) of enzyme structures that degrade, modify, or produce glycosidic bonds^65^. Specifically, the GHs family, which was responsible for the hydrolysis and transglycosylation of glycosidic bonds, was a key enzyme for hydrolyzing complex carbohydrates. Carbohydrate esterase CEs and carbohydrate-binding module CBM promote the actions of GHs on complex polysaccharides by removing lipid-based modifications in polysaccharides or promoting the interactions between enzymes and substrates^66^. In LAB, various kinds of CAZymes participated in the degradation of polysaccharides, like plant cell walls, and resulted in the reduction of neutral detergent fibers after ensiling. Mannose, glucose, xylose, and other glycogens produced provided further glycogens for the growth of LAB; especially, CBM35 and CBM4 in EN5 could further improve the polysaccharide degradation capacity. With the double binding specificity to xylan and mannan, the CBM35 module was a necessary functional structure for bacterial enzymatic hydrolysis of lignocellulosic biomass^67^. CBM4 promoted the pyrolysis of insoluble cellulose xyloglucan by changing the properties of the cellulase catalytic domain^68,69^. The high adaptability of EN5 to the silage environment was attributed to the synergy of this multienzyme participation. Li et al. (2022) showed that the diversity of *L. plantarum* genomes is attributed to its niche specificity, and dairy products and animal-derived isolates have a variety of environment-specific genes^70^. Our analysis also confirmed the polymorphism of the genome of *P. pentosaceus* in different environments. Based on the KEGG annotation of the unique gene of *P. pentosaceus*, it is confirmed that the difference of carbohydrate transport and metabolism mode is the main source of the difference of its genome in different ecological environments. In addition, we found that EN5 contains a unique gene SecB, compared with the genome of other environmental strains of *P. pentosaceus*. It has been reported that SecB protein can enhance the tolerance of microorganisms such as *Escherichia coli* to organic solvents such as organic acids during fermentation^71^, and alleviate the heat stress during the fermentation of *Lactobacillus casei* (current name: *Lacticaseibacillus casei)* by up-regulating the expression of PTS and ABC type transport protein system related to nitrogen and carbon source uptake^72^. Therefore, it was speculated that the existence of SecB protein may promote the absorption and utilization of glycogen in EN5 and alleviate the inhibition of organic acid stress on alfalfa silage fermentation, thus making EN5 strain with higher competition in silage. Therefore, we consider the phyllosphere endophytic microbiome in alfalfa is a potential novel route to enhance silage fermentation quality compared to the tradition phyllosphere epiphytic additive. The current study also deepens the understanding of the niche-specific genome diversity of LAB, but the reason for the diversity of genome and the mechanism of recruitment of endophytes in the phyllosphere are still unknown. Further *in planta* experiments is necessary to explore the restrictive factors of phyllosphere recruitment of endophytic LAB, and how to further affect silage production. Overall, our research confirmed the effect of phyllosphere endophytes in practical agricultural production and provided a new route for developing microbial inoculants for ensiling.

## Methods

### Sample collection

A total of 58 aboveground alfalfa samples were collected from six main alfalfa-producing areas in China (Supplementary Table 8). Every sample included five replicates, and the distance was around 20 meters among replicates. The aboveground parts of the whole alfalfa with a 5–8 cm stubble were cut with gloves and scissors sterilized with alcohol, and placed into sterile sampling bags, before being transported back to the laboratory with ice.

### Phyllosphere bacteria isolation and identification

The 20.0 g whole plant samples from the sampling bags were placed in 250 mL sterile conical flasks and added with 180 mL of sterile saline, sealed and placed in a shaker at 4 °C at a rate of 240 r/min for 2 h, to prepare the suspension liquid of phyllosphere epiphytic microorganisms. To prepare the suspension liquid of phyllosphere endophytic microorganisms, the above whole plant samples were then soaked in 75% ethanol for 90 s, in 3.25% sodium hypochlorite for 120 s and in 75% alcohol for 30 s, and then rinsed in sterile distilled water three times for surface sterilization^73,74^. A final rinsing solution was spread on an LB plate to check whether the microorganisms were removed completely before the further experiments^75^. The sterilized samples were put into sterilized mortars for grinding, then added with 45 mL normal saline, and mixed fully for collection of suspension liquid of phyllosphere endophytic microorganisms.

The suspension liquids were further diluted for culturing. Each aliquot of the diluents (100 μL) was placed on four types of medium (i.e., GS: Gauze’s Synthetic Medium No.1, Qingdao Hope Bio-Technology Co., Ltd., China; MRS: Man Rogosa Sharpe Agar Medium; TSA: Soybean-Casein Digest Agar Medium; LA: LB Nutrient Agar, Beijing AOBOXING Bio-Tech Co., Ltd., China), and cultured at 30 °C. On the 2th and 4th days after culturing, the bacterial colonies were screened, cultured, and purified for further study, according to the parameters of size, shape, edge, luster, texture, color, and transparency.

Glycerol suspension liquids of all pure culture bacteria (15%) were stored in duplicate in the freezer at −80°C. Purified colonies were selected, and the cells were lysed with 5 μL NaOH/SDS lysis buffer (Amresco, USA). Then the lysed solution was further diluted with 100 μL deionized water. Two microliters of the above dilution were used for PCR-based amplification of 16S rRNA gene sequences with KOD Fx DNA polymerase (TOYOBO, Japan) using a KOD-recommended PCR program with 94 °C for 3 min, followed by 30 cycles of denaturing at 94 °C for 30 s, annealing at 55 °C for 30 s and extension at 72 °C for 60 s, and the final extension at 72 °C for 5 min (using the universal bacterial primers: 27F: 5′-AGAGTTTGATCCTGGCTCAG-3′; 1492R: 5′-GGTTACCTTGTTACGACTT-3′). The PCR amplified 16S rRNA gene amplicons were sequenced by BGI Genomics, Shenzhen, China. BLAST^76^ was run using the 16S rRNA gene sequences as queries against the NCBI 16S ribosomal RNA sequences (Bacteria and Archaea) database to search for homologous targets (Supplementary Data 1). The phylogenetic tree was generated using neighbor-joining method in software Mega 11 and viewed in iTOL^77,78^, with 500 replications for bootstrapping.

### Screening and characterization of LAB

All LAB strains identified by 16S rRNA genes and bacteria grown in MRS medium were continue cultured on MRS agar medium, and their morphological characteristics, growth capacity, and acid productivity were determined. The LAB strains with a rapid growth and high acid productivity were selected and tested for physiological and biochemical characteristics^79^. The soluble sugar utilization rate of selected LAB was determined using Biolog AN MicroPlate (Biolog, Hayward, USA). According to the manufacturer’s instruction, all the nutrients and biochemical reagents were pre-filled and dried in 96-well plates, and then, 200 μL equal density of LAB suspension was added. The plates were incubated at 30 °C in the dark for 48 h. The average well color development (AWCD) was calculated to reflect the soluble sugar utilization rate of LAB, according to the following formula: AWCD = [(optical density at 590 nm of each well)- (OD value of the control)]/ (OD value of the control). The comprehensive utilization capacity of total sugars in alfalfa according to the sum of each sugar utilization rate of LAB × corresponding sugar content of alfalfa.

### Silage preparation

Alfalfa was artificially planted and harvested by Linhui Grassland Planting Co., Ltd. in Inner Mongolia Autonomous Region (121°60’ east longitude, 43°60’ north latitude). The harvested alfalfa was divided into two groups: phyllosphere bacteria group (PB, fresh alfalfa) and endophytes group (EN, soaked with 75% alcohol to remove epiphytic communities). Before ensiling, the alfalfa was chopped with a sterile chopper directly in a sterile room with a theoretical length of 2–3 cm. After being mixed, 500 g of chopped alfalfa material was manually placed into polyethylene bags. On the 9th, 30th and 60th days, each of the three treated bags was opened to evaluate the fermented products, chemical component, and microbial community. A total of 18 bags (2 treatment groups × 3 periods × 3 replicates) were filled and kept at the ambient temperature (20–30 °C). The chemical characteristics and microbial count in fresh alfalfa and those after surface sterilization are shown in Supplementary Table 9.

According to the key LAB shown in the co-occurrence network of PB and EN microbial communities and the physiological and biochemical characteristics of LAB we screened, finally we selected eight key LAB strains from phyllosphere bacteria as additive to test their performance in alfalfa silage. The experimental treatments were designed as follows: (i) phyllosphere bacteria group (PB, inoculation with sterile distilled water); (ii) inoculation with *Leuconostoc mesenteroides* subsp. *jonggajibkimchii* (EP1); (iii) inoculation with *L. lactis* (EP2); (iv) inoculation with *L. pentosus* (EP3); (v) inoculation with *Enterococcus faecalis* (EP4); (VI) inoculation with *P. pentosaceus* (EN5); (VII) inoculation with *L. plantarum* (EN6); (VIII) inoculation with *Latilactobacillus graminis* (EN7); (IX) inoculation with *Enterococcus mundtii* (EN8). The inoculum for each treatment was 1 × 10^6^ CFU (colony forming unit)/g fresh matter, with PB as the control and eight selected LAB treatments (EP1, EP2, EP3 and EP4 were from epiphytes, EN5, EN6, EN7, and EN8 were from endophytes). Then 500 g of chopped alfalfa materials was mixed evenly with additives and placed manually into polyethylene bags. A total of 81 bags (9 treatment groups × 3 periods × 3 replicates) were filled and kept at the room temperature (20–30°C). The water-soluble carbohydrates in fresh alfalfa were determined by High Pressure Ion Chromatography (HPIC) as followed. Sugar was separated in silage samples by using PA10 membrane anion exchange resin column (250 × 4 mm) and prepose carbon oxides PA10 protective column (50 × 4 mm). 0.10 g of alfalfa sample was put into 15 mL polypropylene tube with 10 mL of deionized water added. The suspension was obtained after 30-min ultrasonic processing and centrifugation at 8000 rpm for 10 min and was diluted by 100 times before being filtered with a 0.22 μM filter. The extracted sample solution was loaded and determined by amperometry detector in HPIC. On the 7th, 30th and 60th days, each of the three treated bags was opened to evaluate the fermented products, their chemical components, and microbial communities. The chemical characteristics and microbial content of the wilted alfalfa are shown in Supplementary Table 10.

### Fermentation quality analysis

Fresh materials and silage samples (20 g) were mixed with 180 mL sterile water and placed at 4 °C for 6 h. The extract liquid was obtained after filtration with filter paper, and the pH was determined with a glass electrode pH meter (PHS-3C, INESA, Shanghai, China). The extract liquid was filtered with a 0.22 μM microporous filter membrane for the determination of organic acids and ammonia nitrogen. Lactic acid, acetic acid, propionic acid, and butyric acid were determined by high performance liquid chromatography (HPLC, column: Shodex RS Pak KC-811; Showa Denko Co., Ltd., Kawasaki, Japan; detector: DAD, 210 nm, SPD-20A; Shimadzu Co., Ltd, Kyoto, Japan; eluent: 3 mmol L^-1^ HClO4, 10 mL/min; temperature: 50 °C). Ammonia nitrogen content was determined by the colorimetric phenol-hypochlorite method of Broderick and Kang (1980)^24^. About 200 g of fresh materials and silage samples were dried in an oven at 65 °C for 48 h to determine the dry matter content. The dried samples were smashed through a 1 mm sieve for the determination of nutrients. The total nitrogen content was determined by FOSS Kjeltec TM 2300, and the crude protein content was calculated. The contents of neutral detergent fiber and acid detergent fiber were determined by Van Soest’s fiber analysis^80^. The content of water-soluble carbohydrates was determined by anthrone-sulfuric acid colorimetry.

The count of microorganisms was determined by the plate count method. Fresh materials and silage samples (10 g) were mixed with 90 mL sterile water and shook at 4 °C for 120 min for the dilution plating procedure. LAB were counted with MRS agar medium and cultured in an anaerobic incubator at 30 °C for 48 h. Molds and yeasts were counted with rose Bengal agar media and cultured in a constant temperature incubator at 28 °C for 48 h. *Escherichia coli* were counted with eosin methylene blue agar media and cultured in a constant temperature incubator at 37 °C for 48 h. All agar media were produced by Beijing Aobox Biotechnology Co., Ltd. (Beijing, China). The count of culturable microorganisms was calculated in CFU/g of FM (fresh matter).

### PacBio sequencing for microbial community

Approximately 1.0 g sample from each time point of each treatment (9, 30, 60 days for PB and EN group in Tongliao; 7, 30, 60 days for PB, EP2, EP3, EN5, and EN6 group in Cangzhou) was collected to extract total DNA using the E.Z.N.A.® soil DNA Kit (Omega Bio-tek, Norcross, GA, U.S.) according to manufacturer’s instructions. The extracted DNA was checked on 1% agarose gel, and DNA concentration and purity were determined with NanoDrop 2000 UV-vis spectrophotometer (Thermo Scientific, Wilmington, USA). For bacterial community, the bacterial 16 S rRNA gene fragments were amplified using the universal bacterial primers 27 F (5′-AGRGTTYGATYMTGGCTCAG-3′) and 1492 R (5′-RGYTACCTTGTTACGACTT-3′). PCR primers for each sample were barcoded with PacBio barcode sequences. Amplification reactions (20 μL volume) with three replicates consisted of 5 × FastPfu buffer 4 μL, 2.5 mM dNTPs 2 μL, forward primer (5 μM) 0.8 μL, reverse primer (5 μM) 0.8 μL, FastPfu DNA Polymerase 0.4 μL, template DNA 10 ng, and DNase-free water. The PCR amplification was performed as follows: initial denaturation at 95 °C for 3 min, followed by 27 cycles of denaturing at 95 °C for 30 s, annealing at 60 °C for 30 s and extension at 72 °C for 45 s, and the final extension at 72 °C for 10 min (ABI GeneAmp® 9700 PCR thermocyclerm, CA, USA). After electrophoresis, PCR products were purified using the AMPure® PB beads (Pacific Biosciences, CA, USA) and quantified with Quantus™ Fluorometer (Promega, WI, USA). Purified products were pooled in equimolar and DNA library was constructed using the SMRTbell® Express Template Prep Kit 2.0 (Pacific Biosciences, CA, USA) according to PacBio’s instructions. Purified SMRTbell libraries were sequenced on the Pacbio Sequel II System (Pacific Biosciences, CA, USA).

### Bacterial community analysis

PacBio raw reads were processed using the SMRTLink software (Version 8.0) to obtain demultiplexed circular consensus sequence (CCS) with a minimum of three full passes and 99% sequence accuracy. CCS reads were barcode-identified and length-filtered. For bacterial 16S rRNA gene, sequences with a length <1000 or >1800 bp were removed.

Generally, bioinformatic analysis of the microbiota was carried out using the Majorbio Cloud platform^81^ and BMKCloud^82^. The optimized-CCS reads were clustered into operational taxonomic units (OTUs) using UPARSE 7.1 with 97% sequence similarity. The most abundant sequence for each OTU was selected as a representative sequence. The OTU table was manually filtered, by removing chloroplast sequences.

The taxonomy of each OTU representative sequence was analyzed by RDP Classifier version 2.2 against the 16S rRNA gene database (Silva v138) using confidence threshold of 0.7. The metabolic potential of the bacterial community and the composition of functional genes were postulated by assigning 16S rRNA marker gene sequences to KEGG (Kyoto Encyclopedia of Genes and Genomes) annotations of sequenced metagenomic sequences using Tax4Fun^83^.

To minimize the effects of sequencing depth on alpha and beta diversity measurement, the number of 16S rRNA gene sequences from each sample were rarefied. Based on the OTUs, rarefaction curves and alpha diversity indices including observed OTUs, Shannon index were calculated with Mothur v1.30.1. The PERMANOVA test was used to assess the percentage of variation explained by the treatments along with its statistical significance using Vegan v2.5-3 package. The distance-based redundancy analysis was performed using Vegan v2.5-3 package to investigate effect of silage characteristic on silage bacterial community. A random-forest classification was conducted by using R packet random Forest 4.6-14^84^. According to the previous method^85,86^, microbial co-occurrence networks were built by using CoNet module of Cytoscape 3.9.1 and were viewed in Gephi software.

### Genome assembly for the selected endophytes

Colonies of selected LAB (*L. lactis* EP2, *L. pentosus* EP3, *P. pentosaceus* EN5 and *L. plantarum* EN6) were grown in de Man Rogosa and Sharpe (MRS) broth anaerobically at 37 °C. Bacterial DNA was extracted with a commercial DNA extraction kit (OMEGA d3350-02) according to the manufacturer’s instructions. The amount of the extracted genomic DNA was quantified using a TBS-380 fluorometer (Turner BioSystems Inc., Sunnyvale, CA). Fragment libraries (200–300 bp) were constructed only with high-quality DNA (OD260/280 = 1.8–2.0, >6 μg). The genome sequencing is carried out according to the standard protocol provided by Oxford Nanopore Technologies (ONT), including sample quality detection, library construction, library quality detection, and library sequencing. Genome assembly was accomplished based on the filtered reads by Canu v1.5 software, and then circlator v1.5.5 was used to circularize genome assembly. For genome annotation, coding genes prediction was performed by Prodigal v2.6.3. Putative candidates were then analyzed by searching for non-mature mutations and frame-shift mutations using GeneWise v2.2.0. Transfer RNA (tRNA) genes were predicted with tRNAscan-SE v2.0, and Ribosome RNA (rRNA) genes were predicted with Infernal v1.1.3. Repetitive sequences were predicted using RepeatMasker. PhiSpy v2.3 was used for prophage prediction and CRT v1.2 was used for CRISPR identification. IslandPath-DIMOB v0.2 was used to predict genomic island. Software antiSMASH v5.0.0 was used to predict secondary metabolic gene clusters, and PromPredict v1 was used for promoter prediction. For functional annotation, the predicted proteins were blasted (e-value: 1e-5) against Nr, Swiss-Prot, TrEMBL, KEGG, and eggNOG, and Blast2go was used for GO annotation. Furthermore, the genes related to pathogenicity and drug resistance were screened by blast against CAZy, TCDB, CARD, PHI, and VFDB databases. Subcellular localization was detected by SignalP, after transmembrane proteins were filtered by TMHMM.

### Comparative genomics analysis for *P. pentosaceus*

We downloaded 119 genome assemblies of *P. pentosaceus*^87^, removed 30 unknown sources and incomplete genomes, and kept 89 ones (Supplementary Data 3). A comparative analysis was conducted by using BPGA1.3^88^, BPGA runs USEARCH for fastest clustering (using 80% sequence identity cut-off) to obtain the core, accessory, and unique genes. These genes were mapped to KEGG pathways, and percentage frequencies were summarized. The phylogenetic tree was performed based on the selected genomes of *P. pentosaceus*, the clustered output is processed to generate tab delimited gene presence absence binary matrix (pan-matrix) which is then used for pan-genome profile calculations with 20 iterations as well as pan-genome based phylogeny and the phylogenetic tree is constructed using the neighbor-joining method.

### Statistics and reproducibility

For chemical composition, fermentation quality, Shannon index, enzyme activity, and microbial counts results were analyzed using one-way analysis of variance (ANOVA) and two-tailed Welch’s *t*-test. The level of statistical significance was set to *P* < 0.05. Co-occurrence network of microbial communities based on the Spearman correlation > |0.6| and *P* < 0.05. Number of the sample sizes and replicates are described in the legends of each graph.

### Reporting summary

Further information on research design is available in the Nature Portfolio Reporting Summary linked to this article.

### Supplementary information

Supplementary Information

Description of Additional Supplementary Files

Supplementary Data 1

Supplementary Data 2

Supplementary Data 3

Supplementary Data 4

Reporting Summary

## Data Availability

All data supporting the results of this study are provided as “Supplementary Data 1–4”. The 16S rRNA genes for isolated phyllosphere bacteria were provided as “Supplementary Data 1”. The CAZymes genes in endophytic lactic acid bacteria can find in “Supplementary Data 2”. Summary of the 89 *Pediococcus pentosaceus* strains from NCBI can find in “Supplementary Data 3”. The source data behind the graphs in the paper are provided as “Supplementary Data 4”. The PacBio sequencing raw data for microbial community has been submitted to the NCBI database with the accession of PRJNA871981 and the four genome assemblies were deposited in the NCBI GenBank database (EP2 for CP115479.1, EP3 for CP115741.1, EN5 for CP115739.1, and EN6 for CP115480.1).
