# Supplementary material for: Characterization of phyllosphere endophytic lactic acid bacteria reveals a potential novel route to enhance silage fermentation quality
Source: Commun Biol. 2024 Jan 22;7:117. doi: 10.1038/s42003-024-05816-3 (PMC10803313; doi:10.1038/s42003-024-05816-3)
Supplement: Supplementary file 1 — Supplementary Information [file 42003_2024_5816_MOESM1_ESM.pdf]

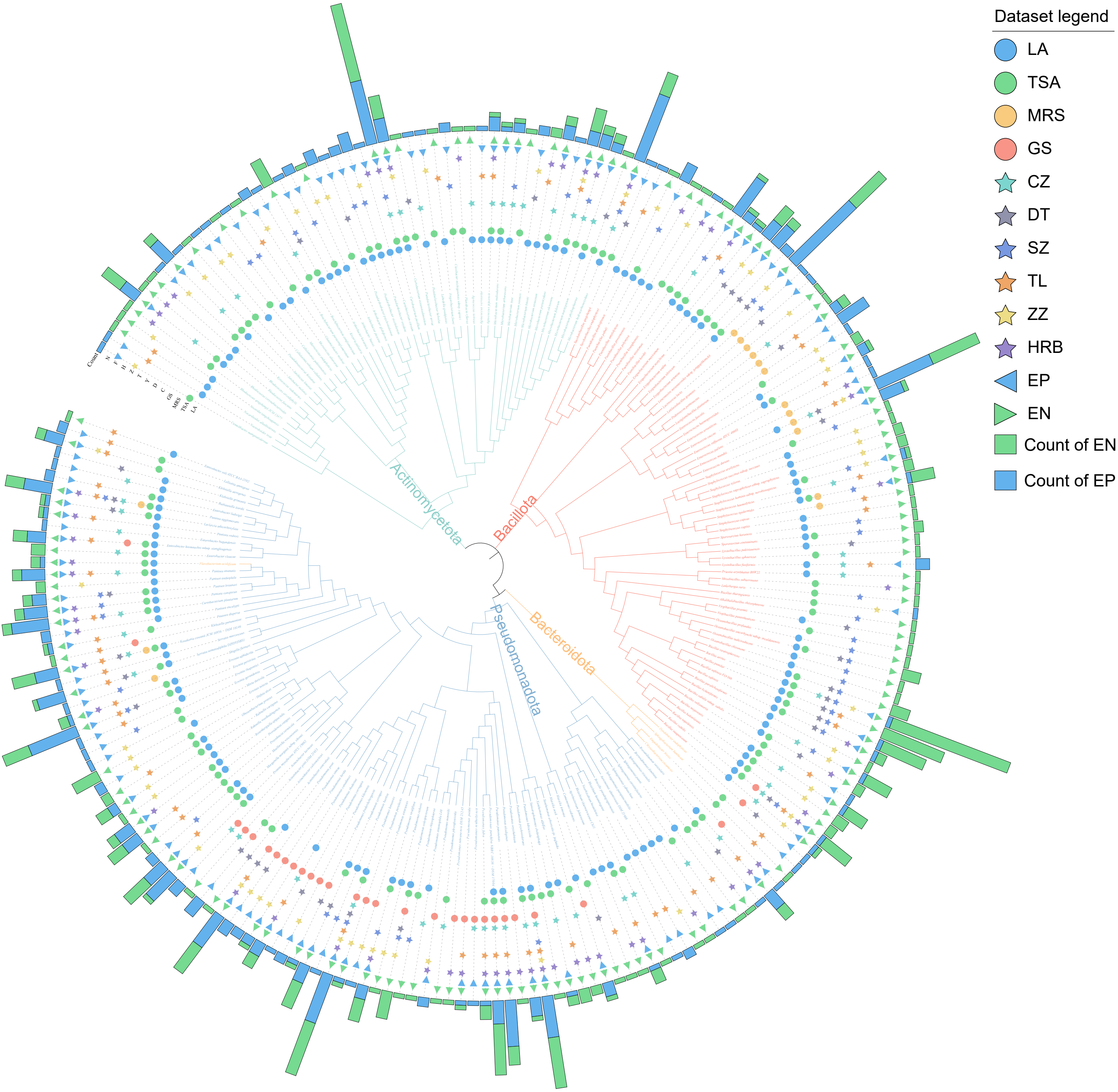

Supplementary Figure 1. Phylogenetic tree based on the 795 culturable phyllosphere bacteria by 16S rRNA gene sequences. The line colors in the inner circle represent four phyla of Bacillota (red), Bacteroidota (orange), Pseudomonadota (blue), and Actinomycetota (green). The colors of outer circles indicate the four media culturable microorganisms were cultured on, i.e., LA: LB nutrient agar; TSA: soybean-casein digest agar medium; MRS: Man Rogosa Sharpe agar medium; GS: Gauze's Synthetic Medium No.1. The stars colors represent the six regions, i.e., CZ: Cangzhou; DT: Datong; HRB: Harbin; TL: Tongliao; SZ: Shuozhou; ZZ: Zhuozhou. The triangle colors represent the endophytes (EN) or epiphytes (EP) isolated from alfalfa leaves, and the bars on the outer represent the microbial abundance.

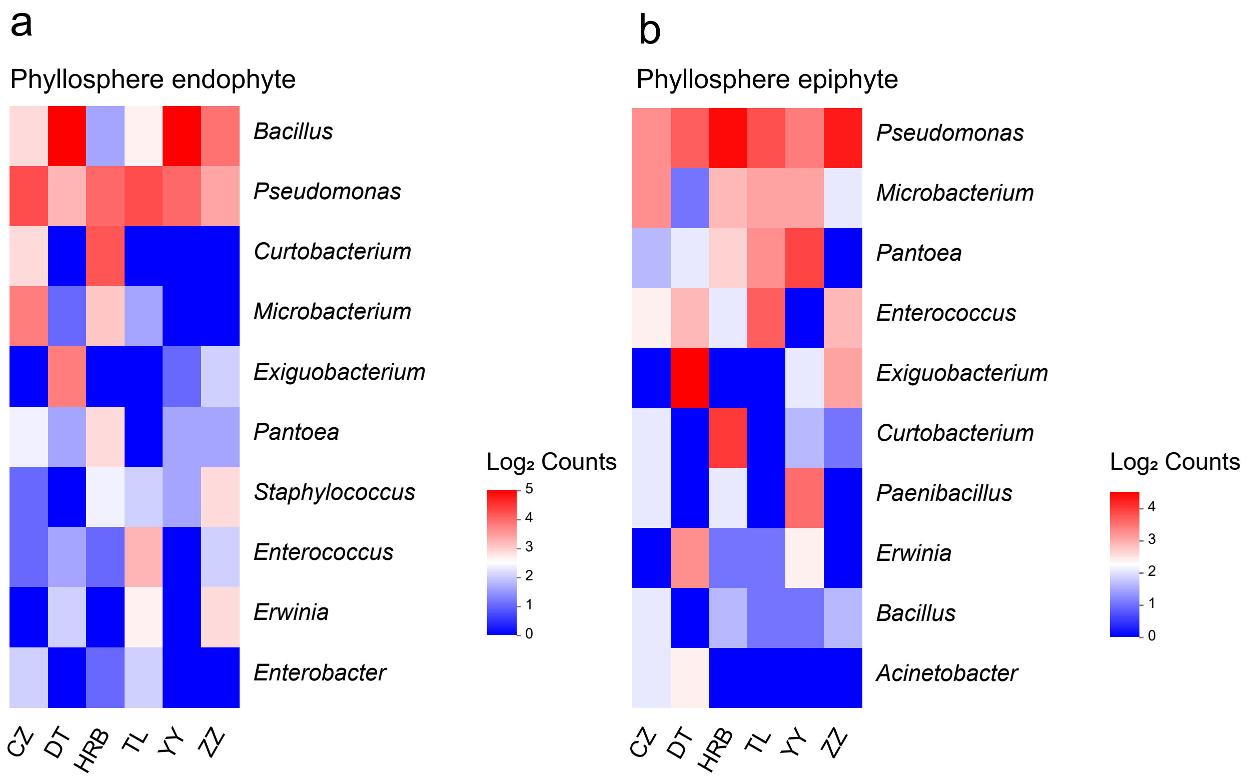

Supplementary Figure 2. The heat map of abundances of phyllosphere endophytes (a) and phyllosphere epiphytes (b) in six different regions. Top 10 genera with relative abundance were shown. CZ: Cangzhou; DT: Datong; HRB: Harbin; TL: Tongliao; SZ: Shuozhou; ZZ: Zhuozhou

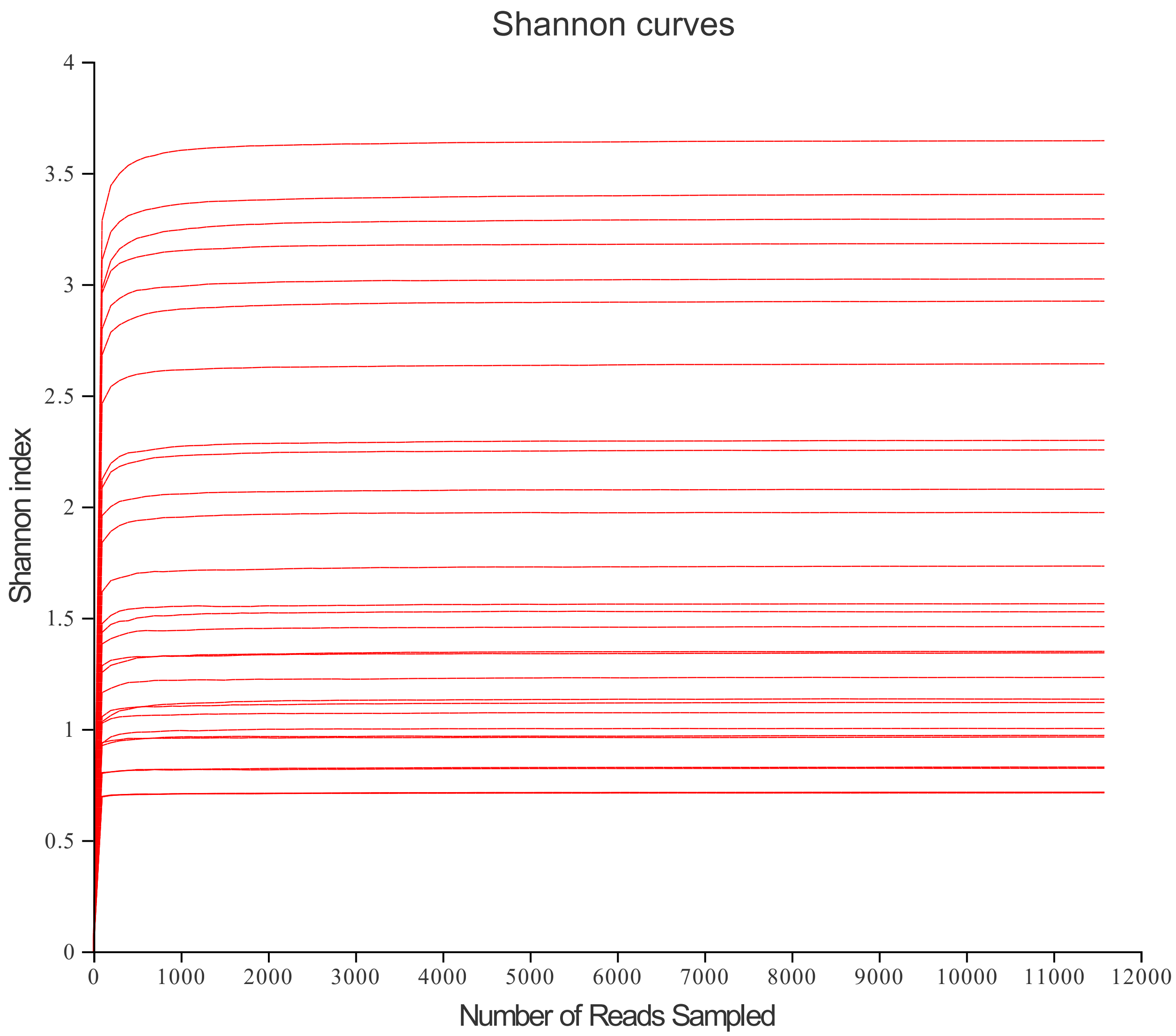

Supplementary Figure 3. Shannon-Wiener rarefaction curve of EN and PB samples. EN: endophytes; PB: naturally silage by phyllosphere bacteria

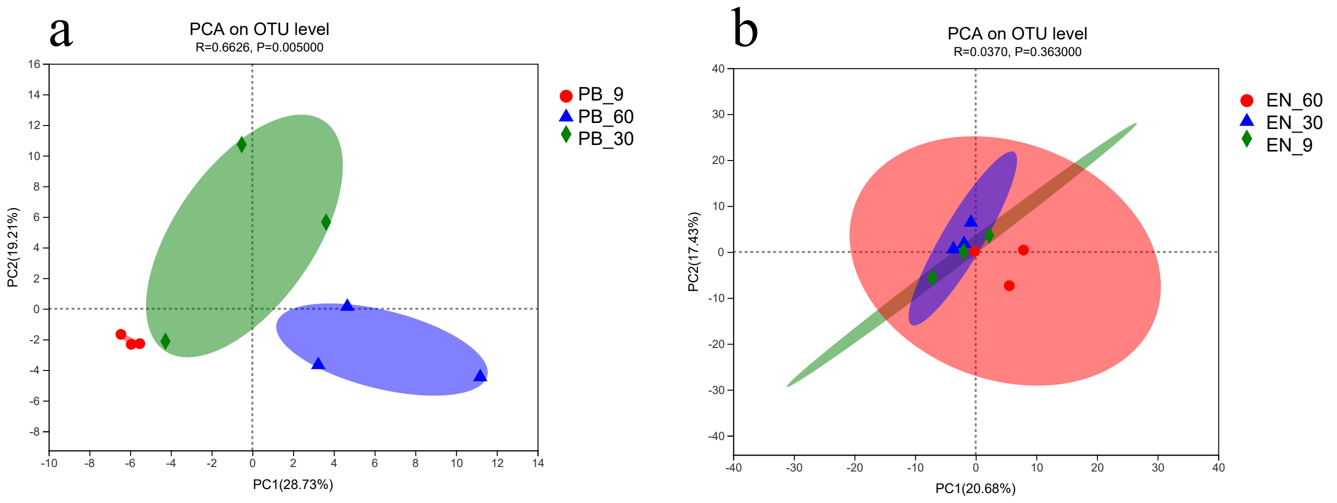

Supplementary Figure 4. PCA plot of beta similarities of bacterial community for PB (a) and EN (b). PB: naturally silage by phyllosphere bacteria; EN: endophytes

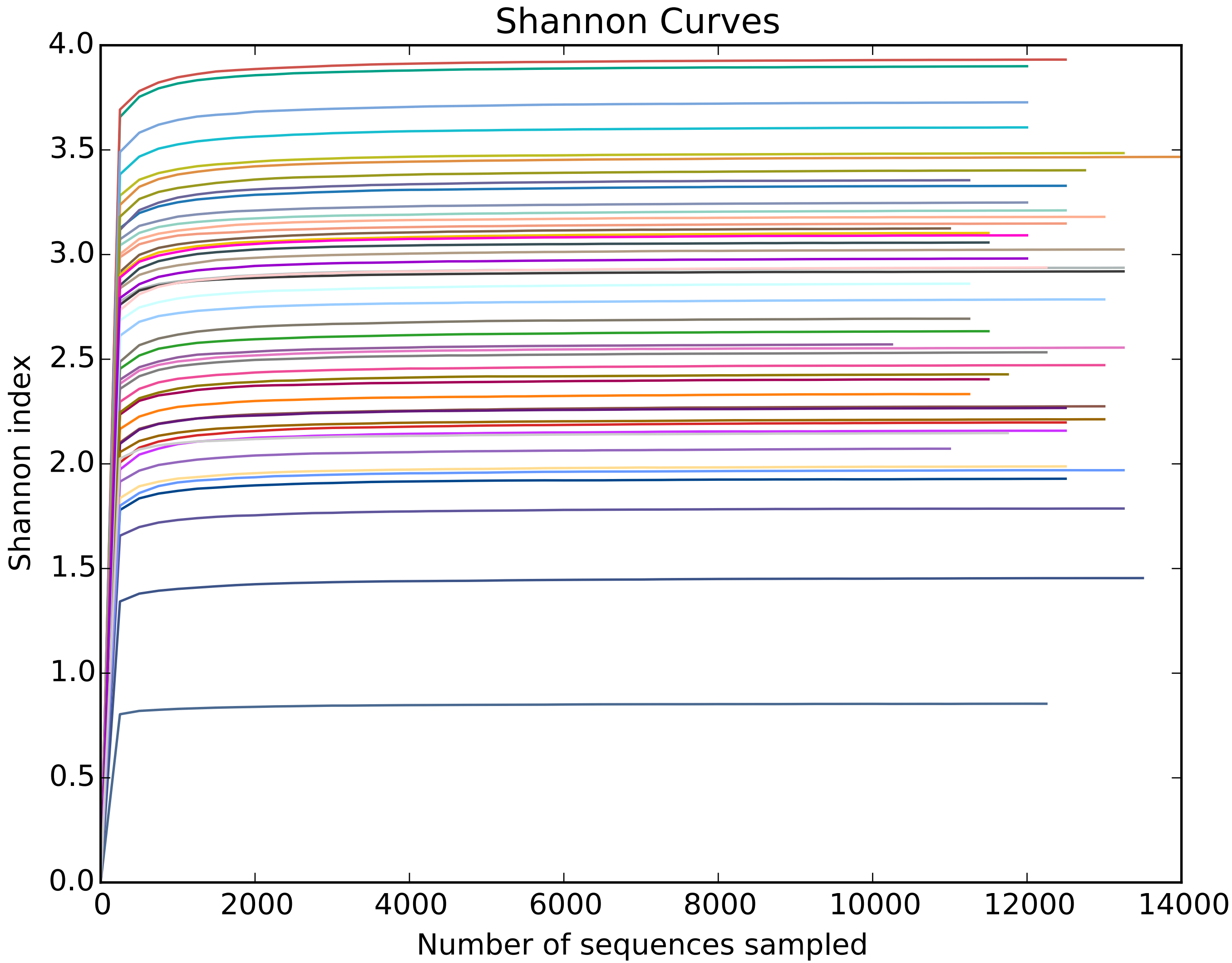

Supplementary Figure 5. Shannon-Wiener rarefaction curve of microbiome sequencing for silage samples with four lactic acid bacteria as additives

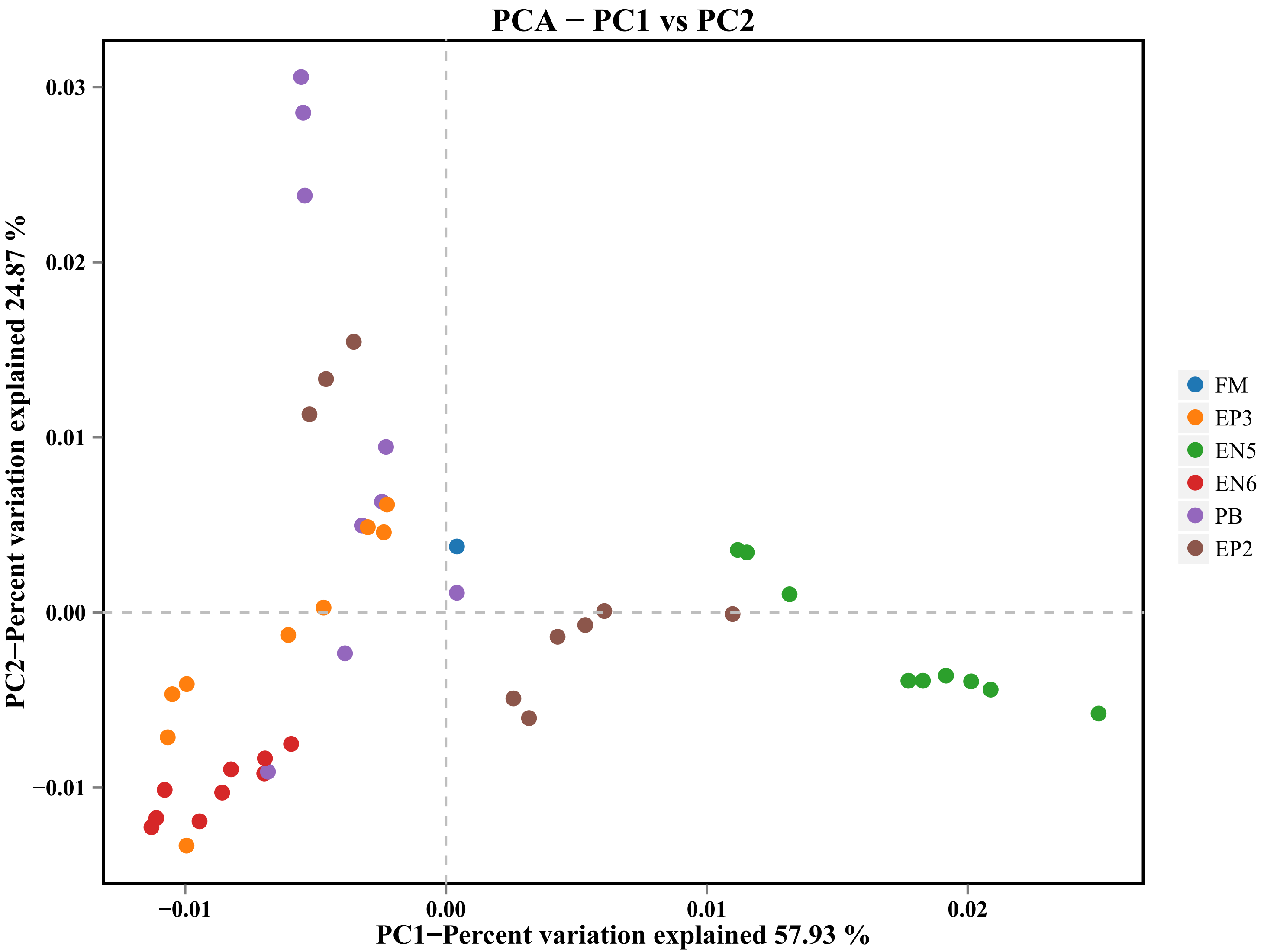

Supplementary Figure 6. PCA plot of beta similarities of bacterial community with endophytic lactic acid bacteria (LAB) as additives. FM: fresh matter of alfalfa; PB: naturally silage by phyllosphere bacteria; EP2: inoculated epiphytic LAB *Lactococcus lactis*; EP3: inoculated epiphytic LAB *Lactiplantibacillus pentosus*; EN5: inoculated endophytic LAB *Pediococcus pentosaceus*; EN6: inoculated endophytic LAB *Lactiplantibacillus plantarum*

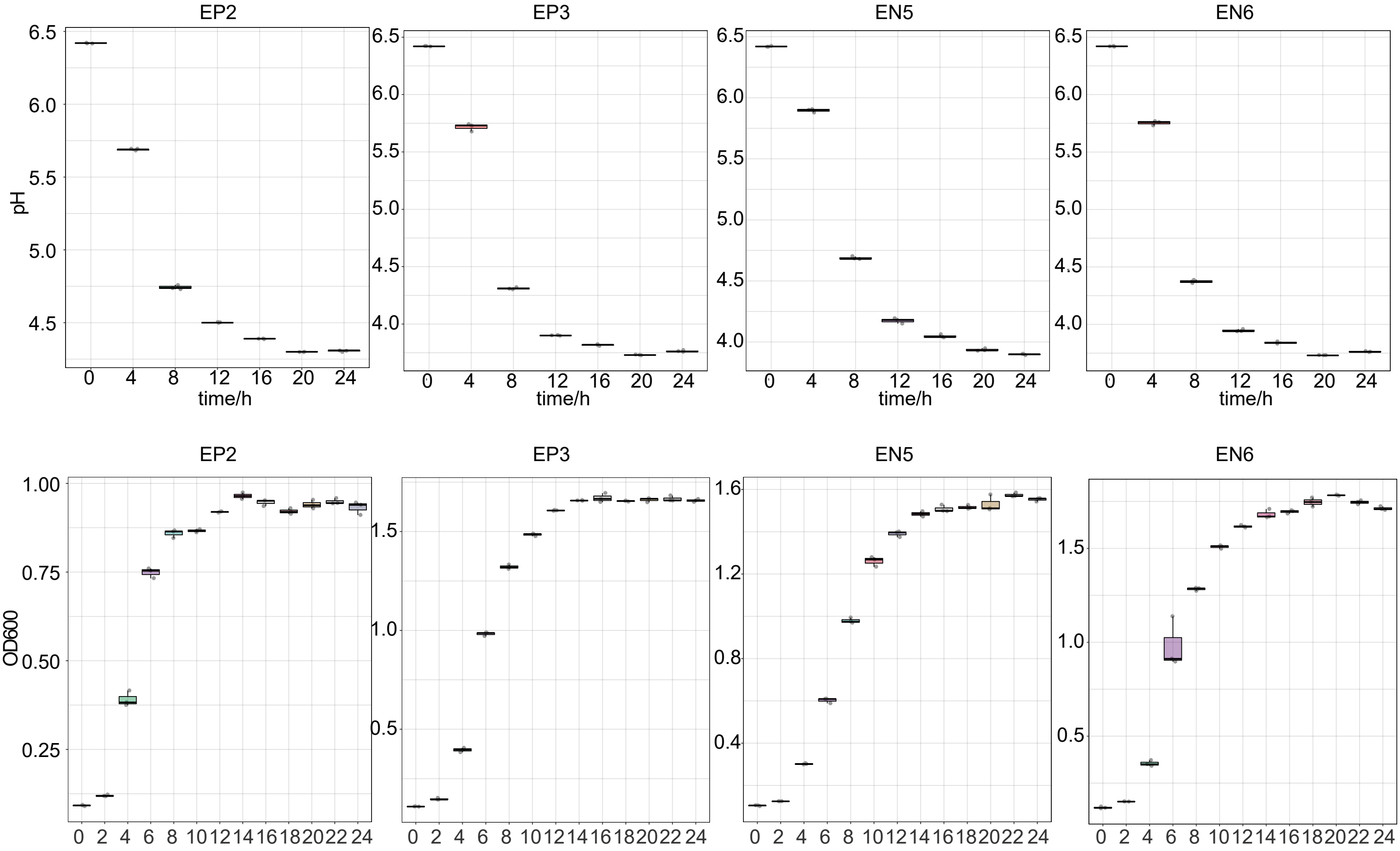

Supplementary Figure 7. Acid production rate and growth curve of lactic acid bacteria (LAB). The box depicts the interquartile range (IQR) between the 25th and 75th percentiles respectively, the top of the box is the first quartile, the bottom is the third quartile, and the line within the box represents the median. The whiskers extend 1.5 times the IQR from the top and bottom of the box, respectively. EP2: epiphytic LAB *Lactococcus lactis*; EP3: epiphytic LAB *Lactiplantibacillus pentosus*; EN5: endophytic LAB *Pediococcus pentosaceus*; EN6: endophytic LAB *Lactiplantibacillus plantarum*

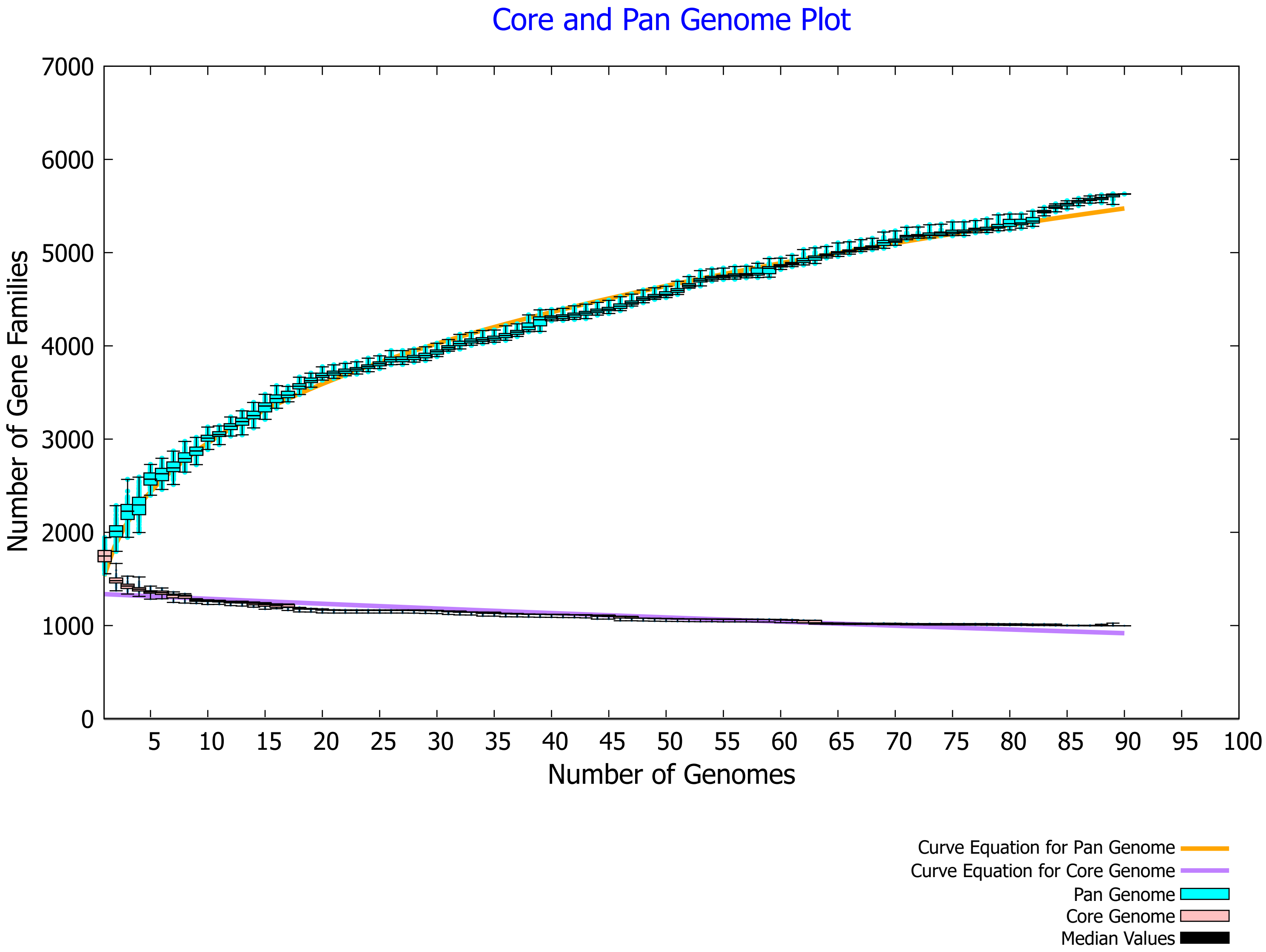

Supplementary Figure 8. Pan-genome and core-genome for *Pediococcus pentosaceus* in comparative genome analysis. The box depicts the interquartile range (IQR) between the 25th and 75th percentiles respectively, the top of the box is the first quartile, the bottom is the third quartile, and the line within the box represents the median. The whiskers extend 1.5 times the IQR from the top and bottom of the box, respectively.

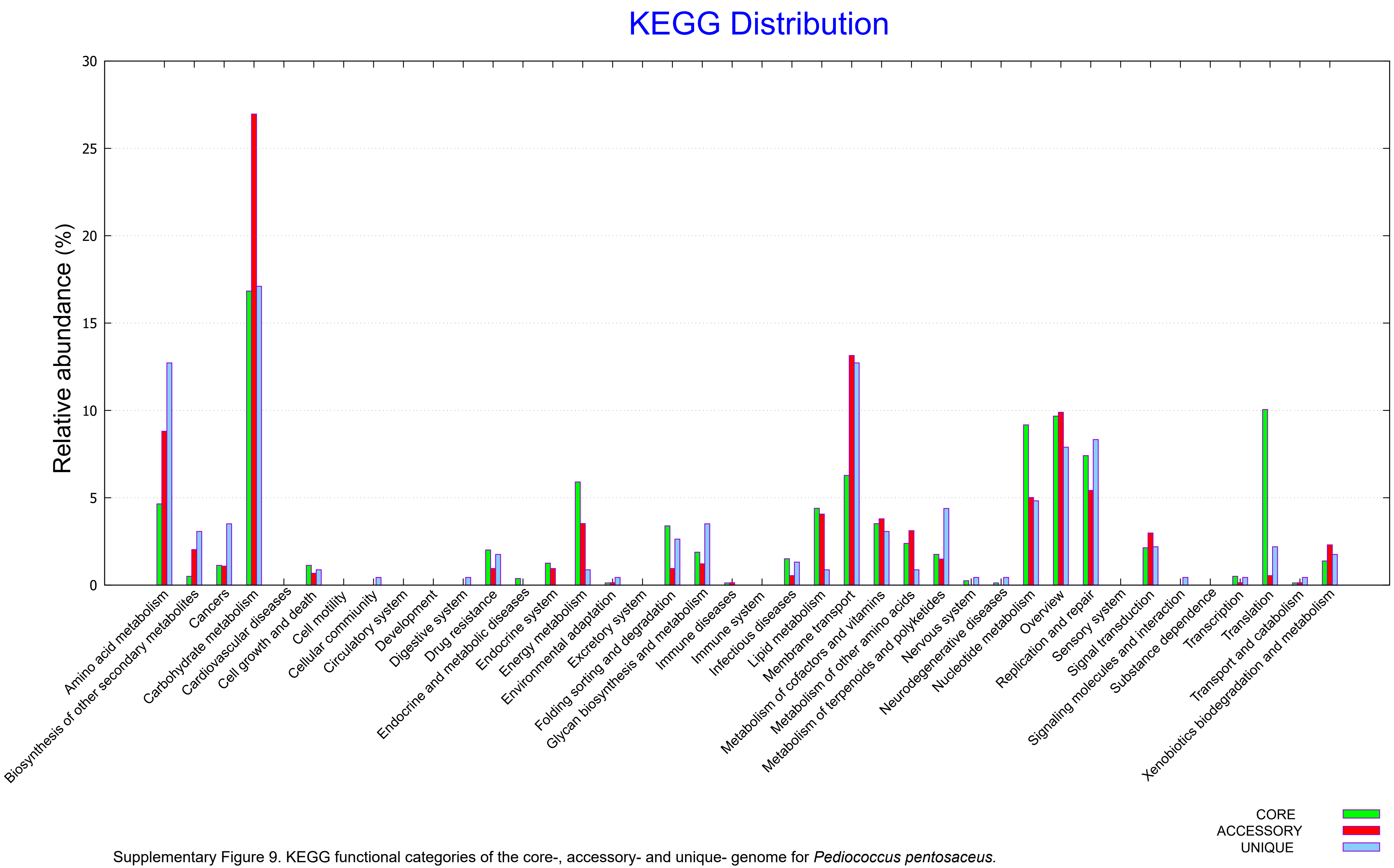

Supplementary Figure 9. KEGG functional categories of the core-, accessory- and unique- genome for *Pediococcus pentosaceus.*

Supplementary Table 1 Effect of PB and EN treatments on fermentation quality, microorganisms, and nutrient composition of alfalfa silage

	T	mean	SEM	*P* value
pH	PB	6.87a	0.125242	4.15405E-08
	EN	5.30b	0.102008	
NH_3-N(g/kg TN)	PB	577.24a	94.96592	0.000163265
	EN	102.42b	20.18533	
LA(g/kg DM)	PB	14.81b	1.602932	0.016184293
	EN	20.69a	1.487434	
AA(g/kg DM)	PB	25.55a	1.960762	9.99373E-05
	EN	14.74b	0.772156	
PA(g/kg DM)	PB	4.91	1.017965	0.795896573
	EN	5.44	1.722628	
BA(g/kg DM)	PB	6.23	2.843463	0.329332869
	EN	2.97	1.545752	
LAB(log_{10}CFU g^{-1} FM)	PB	7.39	0.261109	0.716927723
	EN	7.49	0.121904	
Yeast(log_{10}CFU g^{-1} FM)	PB	3.72a	0.838378	0.003698381
	EN	NDb		
coliform bacteria(log_{10}CFU g^{-1} FM)	PB	5.03a	0.639505	0.000419181
	EN	1.58b	0.789611	
DM(g/kg FM)	PB	215.31b	5.23831	1.015E-05
	EN	250.92a	2.073171	
EE(g/kg DM)	PB	80.36a	4.809717	0.001496094
	EN	60.25b	2.122377	
WSC(g/kg DM)	PB	6.11b	0.416991	0.005486611
	EN	7.84a	0.340936	
CP(g/kg DM)	PB	171.72b	5.018486	6.33619E-06
	EN	212.77a	3.705794	
NDF(g/kg DM)	PB	432.02	10.40959	0.787509036
	EN	428.14	9.598684	
ADF(g/kg DM)	PB	343.04	9.175877	0.179606085
	EN	326	7.951159	

a-b indicates significant difference in different treatment(P<0.05); ND: not detection; T:treatment; TN: total nitrogen; DM: dry matter; LA:lactic acid; AA:acetic acid; PA:propionic acid; BA:butyric acid; FM: fresh matter; WSC: water soluble carbohydrates: NDF: neutral detergent fiber: ADF: acid detergent fibre: CP:crude protein; EE:ether extract; PB: naturally ensilage by phyllosphere bacteria; EN: endophytes.

Supplementary Table 2 Physiological characteristics of selected lactic acid bacteria strains

Number	ph3.0	ph3.5	ph4.0	ph4.5	ph9.0	3%NaCl	6.5%NaCl	4℃	15℃	45℃	OD_{24h}	PH_{24h}	Species name
EP1(Y466)	w	w	w	+	w	+	w	w	+	+	1.106	4.23	*Leuconostoc mesenteroides* subsp. *jonggajibkimchii*
EP2(D456)	w	w	w	w	++	+	+	w	+	+	0.932	4.31	*Lactococcus lactis*
EP3(D450)	w	++	++	+++	w	+++	++	w	++	++	1.657	3.76	*Lactiplantibacillus pentosus*[1]
EP4(T171)	w	w	w	+	++	+	w	w	+	+	0.873	4.64	*Enterococcus faecalis*
EN5(C177)	w	w	+	++	w	++	+	w	++	+	1.552	3.9	*Pediococcus pentosaceus*
EN6(T207)	w	++	++	+++	w	+++	++	w	+++	++	1.712	3.76	*Lactiplantibacillus plantarum*
EN7(Z148)	w	w	+	+	w	++	+	w	++	+	0.992	4.2	*Latilactobacillus graminis*
EN8(T201)	w	w	w	+	+	+	w	w	w	+	0.794	4.6	*Enterococcus mundtii*

w:$OD600_{nm}$ < 0.5; +:0.5≤$OD600_{nm}$ < 1; ++:1≤$OD600_{nm}$ < 1.5; +++： 1.5≤$OD600_{nm}$

[1] Based on the 16S rRNA identification result, D450 was identified as *Lactiplantibacillus plantarum*, and further identified as *Lactiplantibacillus pentosus* after whole genome sequencing

Supplementary Table 3 Effect of four selected lactic acid bacteria inoculants on fermentation quality, microorganisms, and nutrient composition of alfalfa silage

	T	PB	EP2	EP3	EN5	EN6	SEM
LA	7d	25.93c	34.45b	37.95ab	40.52a	37.29ab	5.534
(g/kg DM)	30d	33.93	37.01	45.27	44.21	54.01	
	60d	37.39ab	29.14b	46.03a	48.61a	50.79a	
AA	7d	28.86	32.87	33.36	34.09	32.07	4.603
(g/kg DM)	30d	28.53	25.43	37.79	23.51	39.49	
	60d	29.17ab	38.79a	33.67ab	24.95b	33.09ab	
PA	7d	12.87	12.64	14.46	10.9	12.44	2.667
(g/kg DM)	30d	13.64	11.98	20.16	12.32	18.44	
	60d	15.06	12.94	15.46	12.72	18.18	
BA	7d	ND	ND	ND	ND	ND	
(g/kg DM)	30d	ND	ND	ND	ND	ND	
	60d	ND	ND	ND	ND	ND	
pH	7d	5.69a	5.37b	5.25bc	5.19c	5.24bc	0.036
	30d	5.44a	5.05b	5.05b	4.87c	4.99bc	
	60d	4.91a	4.93a	4.91a	4.72b	4.79ab	
NH_3-N	7d	95.76b	95.74b	116.44a	89.07b	113.31a	8.077
(g/kg TN)	30d	148.72a	134.46ab	150.45a	111.27b	155.76a	
	60d	139.62	146.73	157.08	136.83	161	
LAB	7d	8.03c	8.58abc	8.53abc	8.88a	8.80abA	0.124
(Log_{10} CFU/g FM)	30d	7.82	8.42	7.86	8.4	7.93	
	60d	7.62	7.41	7.26	7.43	7.37	
coliform bacteria	7d	6.64a	5.56b	4.56c	5.55b	5.24bc	0.163
(Log_{10} CFU/g FM)	30d	4.53	ND	ND	ND	ND	
	60d	ND	ND	ND	ND	ND	
Yeast	7d	6.50a	5.30b	4.94b	4.90b	5.26b	0.218
(Log_{10} CFU/g FM)	30d	ND	ND	ND	ND	ND	
	60d	ND	ND	ND	ND	ND	
DM	7d	326.8	330.55	325.91	321.46	327.7	3.293
(g/kg FM)	30d	329.29	331.12	322.09	335.25	330.72	
	60d	342.29a	322.63b	323.66b	327.20b	331.12ab	
WSC	7d	8.15a	7.51ab	6.88ab	6.17b	6.43ab	0.358
(g/kg DM)	30d	6.14	6.11	6.25	6.61	5.81	
	60d	7.25	6.91	6.41	7.25	6.66	
EE	7d	25.24	22.94	31.4	27.51	30.43	1.635
(g/kg DM)	30d	31.08	35.56	30.45	32.45	28.25	
	60d	31.66	31.82	31.13	33.42	35.52	
NDF	7d	381.98	369.98	384.42	384.89	382.02	7.111
(g/kg DM)	30d	360.09	357.49	357.55	365.15	375.38	
	60d	352.65	368.8	369.37	364.74	359.63	
ADF	7d	280.25	271.59	285.98	280.54	280.26	5.976
(g/kg DM)	30d	263.02	266.24	265.41	274.98	283.22	
	60d	264.07	274.04	279.8	269.27	267.88	
CP	7d	238.96	241.81	238.51	246.8	235.78	3.248
(g/kg DM)	30d	235.65	237.59	241.5	239.22	233.76	
	60d	234.41	234.68	238.89	240.12	236.74	

a-c indicates significant difference in different treatment($P<0.05$); ND: not detection; T:treatment; TN: total nitrogen; DM: dry matter; LA:lactic acid; AA:acetic acid; PA:propionic acid; BA:butyric acid; FM: fresh matter; LAB:lactic acid bacteria; CFU:colony forming unit; WSC: water soluble carbohydrates: NDF: neutral detergent fiber: ADF: acid detergent fibre: CP:crude protein; EE:ether extract; PB: naturally ensilage by phyllosphere bacteria; EP2: inoculated epiphytic lactic acid bacteria *Lactococcus lactis*; EP3: inoculated epiphytic lactic acid bacteria *Lactiplantibacillus pentosus*; EN5: inoculated endophytic lactic acid bacteria *Pediococcus pentosaceus*; EN6: inoculated endophytic lactic acid bacteria *Lactiplantibacillus plantarum*.

Supplementary Table 4 Genes related to mannose phosphotransferase in endophytic lactic acid bacteria genomes

#GeneID	nr_annotation
EP2_GE000391	['GE000391', 'mannose-6-phosphate isomerase, class I [Lactococcus lactis]', 'gi|502661419|ref|WP_012897453.1|', '5.6e-187', '100.00', '100.00', '662']
EP2_GE001317	['GE001317', 'PTS mannose transporter subunit IIAB [Lactococcus lactis]', 'gi|489222469|ref|WP_003130898.1|', '1.6e-176', '100.00', '100.00', '627']
EP2_GE001318	['GE001318', 'PTS mannose/fructose/sorbose transporter subunit IIC [Lactococcus lactis]', 'gi|505239784|ref|WP_015426886.1|', '1.7e-136', '100.00', '100.00', '494']
EP2_GE001319	['GE001319', 'PTS mannose/fructose/sorbose transporter family subunit IID [Lactococcus lactis]', 'gi|505239785|ref|WP_015426887.1|', '1.5e-171', '100.00', '100.00', '611']
EP3_GE000440	['GE000440', 'MULTISPECIES: PTS mannose transporter subunit IIAB [Lactobacillus]', 'gi|489733642|ref|WP_003637738.1|', '8.4e-178', '100.00', '100.00', '632']
EP3_GE000441	['GE000441', 'MULTISPECIES: PTS mannose/fructose/sorbose transporter subunit IIC [Lactobacillus]', 'gi|489733643|ref|WP_003637739.1|', '1.1e-135', '99.26', '100.00', '491']
EP3_GE000442	['GE000442', 'PTS mannose/fructose/sorbose transporter family subunit IID [Lactobacillus pentosus]', 'gi|912992418|ref|WP_050339212.1|', '3.0e-172', '100.00', '100.00', '613']
EP3_GE001989	['GE001989', 'mannose-6-phosphate isomerase, class I [Lactobacillus pentosus]', 'gi|1046602893|ref|WP_065674774.1|', '8.8e-196', '100.00', '100.00', '691']
EN5_GE001098	['GE001098', 'PTS mannose/fructose/sorbose transporter subunit IIB [Pediococcus pentosaceus]', 'gi|488923095|ref|WP_002834170.1|', '2.3e-83', '100.00', '100.00', '317']
EN5_GE001100	['GE001100', 'PTS system mannose/fructose/sorbose family transporter subunit IID [Pediococcus pentosaceus]', 'gi|499993139|ref|WP_011673857.1|', '1.5e-143', '100.00', '100.00', '518']
EN5_GE001321	['GE001321', 'PTS mannose transporter subunit IIAB [Pediococcus pentosaceus]', 'gi|488922156|ref|WP_002833231.1|', '1.6e-176', '100.00', '100.00', '627']
EN5_GE001322	['GE001322', 'PTS mannose/fructose/sorbose transporter subunit IIC [Pediococcus pentosaceus]', 'gi|499992989|ref|WP_011673707.1|', '4.7e-134', '100.00', '100.00', '486']
EN5_GE001323	['GE001323', 'PTS mannose/fructose/sorbose transporter family subunit IID [Pediococcus pentosaceus]', 'gi|488922159|ref|WP_002833234.1|', '7.4e-168', '100.00', '100.00', '599']
EN5_GE001326	['GE001326', 'PTS mannose/fructose/sorbose transporter subunit IIB [Pediococcus pentosaceus]', 'gi|488922162|ref|WP_002833237.1|', '2.0e-82', '100.00', '100.00', '314']
EN5_GE001498	['GE001498', 'mannose-6-phosphate isomerase, class I [Pediococcus pentosaceus]', 'gi|499992899|ref|WP_011673617.1|', '2.0e-192', '100.00', '100.00', '680']
EN6_GE000849	['GE000849', 'MULTISPECIES: mannose-6-phosphate isomerase, class I [Lactobacillus]', 'gi|489737333|ref|WP_003641428.1|', '3.4e-195', '100.00', '100.00', '689']
EN6_GE002325	['GE002325', 'MULTISPECIES: PTS mannose transporter subunit IIAB [Lactobacillus]', 'gi|489736813|ref|WP_003640908.1|', '1.4e-169', '100.00', '100.00', '604']
EN6_GE002326	['GE002326', 'MULTISPECIES: PTS mannose/fructose/sorbose transporter subunit IIC [Lactobacillus]', 'gi|489736814|ref|WP_003640909.1|', '8.6e-136', '100.00', '100.00', '492']
EN6_GE002327	['GE002327', 'MULTISPECIES: PTS mannose/fructose/sorbose transporter family subunit IID [Lactobacillus]', 'gi|489736815|ref|WP_003640910.1|', '3.0e-172', '100.00', '100.00', '613']
I6G30_RS06430	mannose-6-phosphate isomerase [Pediococcus pentosaceus]
I6G30_RS07365	PTS mannose transporter subunit IIAB [Pediococcus pentosaceus]
I6G30_RS07355	PTS mannose transporter subunit IID [Pediococcus pentosaceus]
I6G30_RS01110	PTS mannose transporter subunit IID [Pediococcus pentosaceus]
I6G30_RS01100	PTS mannose transporter subunit IID [Pediococcus pentosaceus]
I6G30_RS08520	PTS mannose transporter subunit IID [Pediococcus pentosaceus]

Supplementary Table 5 Core-, accessory-, exclusively absent and unique genes in *Pediococcus pentosaceus* strains

Genome no.	Organism name	No. of core genes	No. of accessory genes	No. of unique genes	No. of exclusively absent genes
1	EN5.faa	998	752	9	0
2	GCA_001411765.2.faa	998	665	3	4
3	GCA_002173535.1.faa	998	855	91	9
4	GCA_002202155.1.faa	998	750	4	0
5	GCA_002982155.1.faa	998	635	3	4
6	GCA_003429405.1.faa	998	685	6	0
7	GCA_004115445.1.faa	998	655	34	6
8	GCA_005864405.1.faa	998	815	22	0
9	GCA_006770865.1.faa	998	638	2	1
10	GCA_007923185.1.faa	998	750	26	2
11	GCA_007992275.1.faa	998	659	15	2
12	GCA_009791435.1.faa	998	715	14	5
13	GCA_009808085.1.faa	998	808	11	0
14	GCA_009808645.1.faa	998	778	29	4
15	GCA_009808655.1.faa	998	726	0	0
16	GCA_009808815.1.faa	998	634	43	1
17	GCA_009808845.1.faa	998	890	9	0
18	GCA_009809195.1.faa	998	675	18	3
19	GCA_009809425.1.faa	998	687	7	1
20	GCA_009809485.1.faa	998	651	1	0
21	GCA_009809595.1.faa	998	826	7	0
22	GCA_009809665.1.faa	998	899	16	0
23	GCA_009809875.1.faa	998	654	1	0
24	GCA_009913955.1.faa	998	710	19	0
25	GCA_009913995.1.faa	998	781	0	0
26	GCA_009914015.1.faa	998	784	25	0
27	GCA_009914035.1.faa	998	782	0	0
28	GCA_009930955.1.faa	998	602	8	1
29	GCA_011009615.1.faa	998	725	22	4
30	GCA_013385165.1.faa	998	806	27	3
31	GCA_015612965.1.faa	998	832	14	0
32	GCA_015612975.1.faa	998	703	8	1
33	GCA_015613005.1.faa	998	773	0	0
34	GCA_015613015.1.faa	998	744	7	1
35	GCA_015613035.1.faa	998	745	3	0
36	GCA_015613055.1.faa	998	754	28	2
37	GCA_015613065.1.faa	998	904	6	1
38	GCA_015613105.1.faa	998	814	109	1
39	GCA_015613115.1.faa	998	732	0	0
40	GCA_015613165.1.faa	998	621	8	1
41	GCA_015613205.1.faa	998	678	1	2
42	GCA_015613235.1.faa	998	640	7	1
43	GCA_015613245.1.faa	998	647	3	0
44	GCA_015613265.1.faa	998	731	24	10
45	GCA_015613305.1.faa	998	683	7	0
46	GCA_015613315.1.faa	998	661	42	12
47	GCA_015613745.1.faa	998	775	33	1
48	GCA_015613765.1.faa	998	737	23	2
49	GCA_015613785.1.faa	998	749	13	2
50	GCA_016921135.1.faa	998	638	19	1
51	GCA_019008315.1.faa	998	861	1	1
52	GCA_019614475.1.faa	998	676	43	2
53	GCA_019793535.1.faa	998	640	11	0
54	GCA_020882545.2.faa	998	741	0	0

Genome no.	Organism name	No. of core genes	No. of accessory genes	No. of unique genes	No. of exclusively absent genes
55	GCA_021378055.1.faa	998	724	13	1
56	GCA_022394815.1.faa	998	868	3	0
57	GCA_022678665.1.faa	998	740	0	0
58	GCA_022690785.1.faa	998	717	58	1
59	GCA_023277765.1.faa	998	673	3	1
60	GCA_023277785.1.faa	998	795	3	5
61	GCA_023277805.1.faa	998	854	17	1
62	GCA_023369775.1.faa	998	633	58	28
63	GCA_023740675.1.faa	998	818	8	1
64	GCA_023743315.1.faa	998	549	9	1
65	GCA_023743335.1.faa	998	830	26	1
66	GCA_023744045.1.faa	998	790	18	0
67	GCA_023744355.1.faa	998	807	15	0
68	GCA_024349305.1.faa	998	588	6	0
69	GCA_024539715.1.faa	998	703	57	2
70	GCA_024580495.1.faa	998	797	10	0
71	GCA_024580845.1.faa	998	771	3	0
72	GCA_024622005.1.faa	998	592	0	0
73	GCA_025122005.1.faa	998	728	0	0
74	GCA_025122025.1.faa	998	729	1	0
75	GCA_025122095.1.faa	998	747	27	0
76	GCA_025122105.1.faa	998	732	1	0
77	GCA_025122115.1.faa	998	649	12	0
78	GCA_025122155.1.faa	998	726	9	0
79	GCA_025122175.1.faa	998	730	0	0
80	GCA_025122195.1.faa	998	774	30	2
81	GCA_025132875.1.faa	998	700	112	0
82	GCA_025132915.1.faa	998	627	59	13
83	GCA_025188055.1.faa	998	852	33	0
84	GCA_025188065.1.faa	998	659	11	1
85	GCA_025188105.1.faa	998	609	7	0
86	GCA_025188125.1.faa	998	754	26	0
87	GCA_025188145.1.faa	998	621	3	3
88	GCA_025770455.1.faa	998	741	26	0
89	GCA_025770465.1.faa	998	777	32	0
90	GCA_025770495.1.faa	998	764	34	1

Supplementary Table 6 Unique genes in EN5 compared with other strains of *Pediococcus pentosaceus*

>unique/1745/1/Org1_Gene72/product="hypothetical protein"
MARQAISFKYFVPKLSYANNGQLYNWDMQDFLDYTLMHRREIRLAVPLGDEIADFEWPDVAYDEVNQLYRFRLSKLRSKNIPARKRVNTPKDDIILAEDEFLGEFNLLIFVPTTG
VLIVQSNFYGLTTKQTELALTGMRNEWKRAINEEEDDMGLVSLDPIPDERAIDRARNAEIYRSFKLRCSNVNTFIDQNFNSDLLSAAVHQTDTLQGHNIEISVTMGGQGSRNET
LGNDDVRAVIDDIQYLRQQNFDVSMHIATKQDEEHKVEFIDLISPVYRTNLVLEIEDRTTIGSEYLYQNYLELNYFDADIHAQNTLNHLLQRN*
>unique/3081/1/Org1_Gene224/product="hypothetical protein"
MDFYHSTTFKSRQLIIKENNLRKTEYDFPHYLDVIFNYYDSNKKIDFLPDVIQGQRQVPYLGDGVYCFDNKQAALNYNEETFITIHCDKPNILDVDEPEFKLALYEFFAQKLMDVV
KEKFYDEDMKQGYAYLSEYLLNKLYDIEDCSSATKFPYENFALMLFLYFTFEKHNEIPDVLCKNFPENPYLYYNIVNTNIIKEIS*
>unique/3365/1/Org1_Gene73/product="hypothetical protein"
MRSYIKRLKEVFKKGNSIDKFNIINYAVFFCFSIVGIFFYCKGYLKSIRFGISDTIAVSSIILGILGVFIGILIGQRENSKFFKATQRNDLGKNFFVKLMAKIRNQFFYNIVFIVFTLLCDFLP
VGINSVLKIFFLAIWFWLFMVILWGVFYIVSVIVDISINDTNMNDRDEPKRN*
>unique/3801/1/Org1_Gene75 /product="PDDEXK_5"
MSTYEKHINGTVSELLAEAFFVSKGYVVSKPINDFNEYDLIIDCEDGLKRVQVKTIYWDNTKMRNMASCVTSHIRGNNQRYNKKYNKKSFDILCAVHKDTKSFYLIPFENIKGRRSI
TFYPDGKPKTVNSRYADFEAYREVL*
>unique/3888/1/Org1_Gene222/product="SecB"
MTKNPKPVINLESYKIADLRYTKDEDEIKKYKSDFEFTPTLAFSKDKKVAKLTIRTSIKINKNKYIEDCAVSLILNGFFEISDDIKNDEKKVASLVITNGTAILFPYVRSIVSMVSGLDSNQ
TILLPTINTTKLFNN*
>unique/3891/1/Org1_Gene223/product="hypothetical protein"
MDKLELFNKELSKKIKENKSFYEKATKELHEKNAFRKFNFIVRQYLKINFSLQHSTKPRLSQSMDSIEPNSTELFMQINVMEFDKGDNNKINKKYIEQDVKAEGSAFFLWYNSKIDG
GEYCDKKSKTSYKPRELQNC*
>unique/3944/1/Org1_Gene1608/product="hypothetical protein"
MGIEPTRAGATIRCVNHFANTAIMAGIVGVEPTLTVLETVVLPLNYIPIIGKNKTKCELNVHYGAGQNRTADTWSFNPLLYRLSYRAKKYINVTTVLTRLELVISCVTGRRPNQLDH
RTKEKILEWRIQGSNL*
>unique/4673/1/Org1_Gene74/product="Terminase_2"
MSKKLTPKQKKFADNYIESGNATQSAIKAGYSKKTAKSVGSENLTKPDIKAYIDKKMREIESDRIMGAQEALEFLTNVVRGEEQTQKKSGGDT*
>unique/5437/1/Org1_Gene254/product="hypothetical protein"
MRLPSYVVLMITLLGCYFAYITGVAKLAEVGILFFMVFAVIALEEICVKLKERS*

Supplementary Table 7 Antibiotic resistance gene abundance statistics of EN5

#gene_ID	ARO_accession	ARO_name	ARO_description	Resistance	Resistance Mechanism	CDS sequence
GE000370	ARO:3001806	OXA-224	OXA-224 is a beta-lactamase found in Pseudomonas aeruginosa	penam; cephalosporin	antibiotic inactivation	ATGGAATTAAGAGGAAAACAAAAAAGATATCTTAGAGCTCGAGCACATTCATACCGCCCAGTTTTTTCCGTGGGTAAAAACGGTCTAACTGAAGCATGGCTCAATCAATTGGCAGGAGCTTTAGACAACCATGAATTATTAAAAATTAATATCCAACAAAATTCAGATGCAACAACGGCTGAAGCTAAAGAATTTATTGAATCTAATACAGATATTCAAGTAGTTCAAACCATTGGACGGGTACTAGTGTTGTTTAAAGTTTCTTCGGAAACGGATAAACGTGAAATTTCAGATACAGTTAAGAAGATATAG

Supplementary Table 8 Geographic locations of samples.

sample name	sample point	City	Province/Autonomous Region	Latitude and longitude
CZ	Huanghua Fengmaoshengyuan Agricultural Technology	Cangzhou	Hebei	N38.29, E117.49
DT	Fengzhuang Village	Datong	Shanxi	N39.99, E113.30
HRB	National Modern Agriculture Demonstration Zone of Heilongjiang Academy of Agricultural Sciences	Harbin	Heilongjiang	N45.84, E126.85
SZ	Yangzhuang Village	Shuozhou	Shanxi	N39.91, E112.44
TL	Linhui Grass Industry	Tongliao	Inner Mongolia Autonomous Region	N43.60, E121.60
ZZ	Zhuozhou Experimental Station of China Agricultural	Zhuozhou	Hebei	N39.46, E115.85

Supplementary Table 9 Characteristics of the PB and EN alfalfa before ensiling

Item	Naturally ensilage by phyllosphere bacteria(PB)	Endophytes(EN)
pH	6.27	6.34
WSC(g/kg DM)	29.27	29.72
EE(g/kg DM)	61.76	60.71
NDF(g/kg DM)	355.48	384.34
ADF(g/kg DM)	267.97	281.3
CP(g/kg DM)	240.4	224.87
DM(g/kg FM)	257.62	264.96
LAB (Log_{10} CFU/g FM)	4.6	3.54
Aerobic bacteria (Log_{10} CFU/g FM)	8.56	4.22
Yeast (Log_{10} CFU/g FM)	5.85	3.85

FM:fresh matter: DM:dry matter: WSC: water soluble carbohydrates: NDF: neutral detergent fiber: ADF: acid detergent fibre; CP:crude protein; EE:ether extract; LAB:lactic acid bacteria; CFU:colony forming unit

Supplementary Table 10 Characteristics of alfalfa before ensiling with endophytic lactic acid bacteria as additive

Items	Fresh matter of alfalfa (FM)
LAB (Log_{10} CFU/g FM)	6.61
coliform bacteria (Log_{10} CFU/g FM)	7.9
Yeast (Log_{10} CFU/g FM)	8.28
WSC (g/kg DM)	23.1
EE (g/kg DM)	24.64
NDF (g/kg DM)	394.43
ADF (g/kg DM)	280.83
CP (g/kg DM)	244.59
DM (g/kg FM)	356.66

FM:fresh matter: DM:dry matter: WSC: water soluble carbohydrates: NDF: neutral detergent fiber: ADF: acid detergent fibre; CP:crude protein; EE:ether extract; LAB:lactic acid bacteria; CFU:colony forming unit
