# Supplementary material for: Characterization of phyllosphere endophytic lactic acid bacteria reveals a potential novel route to enhance silage fermentation quality
Source: Commun Biol. 2024 Jan 22;7:117. doi: 10.1038/s42003-024-05816-3 (PMC10803313; doi:10.1038/s42003-024-05816-3)
Supplement: Supplementary file 2 — Description of Additional Supplementary Files [file 42003_2024_5816_MOESM2_ESM.pdf]

Description of Additional Supplementary Files

File name: Supplementary Data 1
Description: The 16S rRNA gene sequencing analysis for the culturable phyllosphere bacteria in fresh alfalfa.

File name: Supplementary Data 2
Description: The CAZymes genes in endophytic lactic acid bacteria.

File name: Supplementary Data 3
Description: Summary of the 89 *Pediococcus pentosaceus* strains from NCBI.

File name: Supplementary Data 4
Description: The source data behind the graphs in the paper.
