# Supplementary material for: Characterization of phyllosphere endophytic lactic acid bacteria reveals a potential novel route to enhance silage fermentation quality
Source: Commun Biol. 2024 Jan 22;7:117. doi: 10.1038/s42003-024-05816-3 (PMC10803313; doi:10.1038/s42003-024-05816-3)
Supplement: Supplementary file 7 — Reporting Summary [file 42003_2024_5816_MOESM7_ESM.pdf]

nature portfolio

Corresponding author(s): Kuikui Ni

Last updated by author(s): Dec 21, 2023

Reporting Summary

Nature Portfolio wishes to improve the reproducibility of the work that we publish. This form provides structure for consistency and transparency in reporting. For further information on Nature Portfolio policies, see our Editorial Policies and the Editorial Policy Checklist.

Statistics

For all statistical analyses, confirm that the following items are present in the figure legend, table legend, main text, or Methods section.

n/a	Confirmed	
☐	☒	The exact sample size (*n*) for each experimental group/condition, given as a discrete number and unit of measurement
☐	☒	A statement on whether measurements were taken from distinct samples or whether the same sample was measured repeatedly
☐	☒	The statistical test(s) used AND whether they are one- or two-sided<br>*Only common tests should be described solely by name; describe more complex techniques in the Methods section.*
☒	☐	A description of all covariates tested
☐	☒	A description of any assumptions or corrections, such as tests of normality and adjustment for multiple comparisons
☐	☒	A full description of the statistical parameters including central tendency (e.g. means) or other basic estimates (e.g. regression coefficient) AND variation (e.g. standard deviation) or associated estimates of uncertainty (e.g. confidence intervals)
☐	☒	For null hypothesis testing, the test statistic (e.g. *F*, *t*, *r*) with confidence intervals, effect sizes, degrees of freedom and *P* value noted<br>*Give P values as exact values whenever suitable.*
☒	☐	For Bayesian analysis, information on the choice of priors and Markov chain Monte Carlo settings
☒	☐	For hierarchical and complex designs, identification of the appropriate level for tests and full reporting of outcomes
☐	☒	Estimates of effect sizes (e.g. Cohen's *d*, Pearson's *r*), indicating how they were calculated

Our web collection on statistics for biologists contains articles on many of the points above.

Software and code

Policy information about availability of computer code

Data collection	No specialized software was used for data collection.
Data analysis	Sequence analysis was performed using BLAST (https://blast.ncbi.nlm.nih.gov/Blast.cgi), SMRTLink software (Version 8.0). Bioinformatic analysis of the microbiota was carried out using the UPARSE 7.1, RDP Classifier version 2.2, Tax4Fun, Mothur v1.30.1, Vegan v2.5-3 package, R packet random Forest 4.6-14, Cytoscape 3.9.1. Genome assembly was accomplished based on the filtered reads by Canu v1.5, circlator v1.5.5, Prodigal v2.6.3, GeneWise v2.2.0, tRNAscan-SE v2.0, Infernal v1.1.3, RepeatMasker. PhiSpy v2.3, CRT v1.2, IslandPath-DIMOB v0.2, antiSMASH v5.0.0, PromPredict v1, TMHMM. Comparative genomics analysis was performed using BPGA1.3. Statistical analysis was using SPSS 17.0.

For manuscripts utilizing custom algorithms or software that are central to the research but not yet described in published literature, software must be made available to editors and reviewers. We strongly encourage code deposition in a community repository (e.g. GitHub). See the Nature Portfolio guidelines for submitting code & software for further information.

Data

Policy information about availability of data

All manuscripts must include a data availability statement. This statement should provide the following information, where applicable:

- Accession codes, unique identifiers, or web links for publicly available datasets
- A description of any restrictions on data availability
- For clinical datasets or third party data, please ensure that the statement adheres to our policy

All data supporting the results of this study are provided as “Supplementary Data 1-4”. The 16S rRNA genes for isolated phyllosphere bacteria were provided as “Supplementary Data 1”. The CAZymes genes in endophytic lactic acid bacteria can find in “Supplementary Data 2”. Summary of the 89 Pediococcus pentosaceus strains from NCBI can find in “Supplementary Data 3”. The source data behind the graphs in the paper are provided as “Supplementary Data 4”. The PacBio sequencing raw data for microbial community has been submitted to the NCBI database with the accession of PRJNA871981 and the four genome assemblies were deposited in the NCBI GenBank database (EP2 for CP115479.1, EP3 for CP115741.1, EN5 for CP115739.1, and EN6 for CP115480.1).

Human research participants

Policy information about studies involving human research participants and Sex and Gender in Research.

Reporting on sex and gender	No human research participants were involved in the study.
Population characteristics	No human research participants were involved in the study.
Recruitment	No human research participants were involved in the study.
Ethics oversight	No human research participants were involved in the study.

Note that full information on the approval of the study protocol must also be provided in the manuscript.

Field-specific reporting

Please select the one below that is the best fit for your research. If you are not sure, read the appropriate sections before making your selection.

☐ Life sciences ☐ Behavioural & social sciences ☒ Ecological, evolutionary & environmental sciences

For a reference copy of the document with all sections, see nature.com/documents/nr-reporting-summary-flat.pdf

Ecological, evolutionary & environmental sciences study design

All studies must disclose on these points even when the disclosure is negative.

Study description	In order to explore the diversity of alfalfa phylosphere microbiota， A total of 58 aboveground alfalfa samples were collected from six main alfalfa-producing areas in China. Every sample included five replicates, and the distance was around 20 meters among replicates, a total of 795 cultivable alfalfa phylosphere microorganisms were obtained. Further , the harvested alfalfa was divided into two groups: phyllosphere bacteria group (PB) and endophytes group (EN) to exploration the effects of phylosphere bacteria fermentation and endophytic fermentation on the quality of alfalfa silage, a total of 18 bags (2 treatment groups×3 periods×3 biologically independent samples) were filled and identified. Finally, a total of 81 bags (9 treatment groups×3 periods×3 biologically independent samples) of alfalfa were filled and identified to evaluation of the application effects of Endophytic and epiphytic lactic acid bacteria additives.
Research sample	Alfalfa raw materials come from five different regions in China and are fermented to produce silage samples, all alfalfa samples are from the budding stage to the early blooming stage and no pests or diseases. The microorganisms involved are all isolationed from these alfalfa samples.
Sampling strategy	No statistical-based sample size caculation was performed. In order to perform t-test, we set at least 3 biological replicates for each treatment group.
Data collection	Data collected from the Forage Production, Processing and Utilization Laboratory of China Agricultural University. The genome sequence used for comparison comes from NCBI
Timing and spatial scale	Samples were collected from the April 2021 to October 2021, Datasets were collected from the December 2022 release of NCBI.
Data exclusions	No data were excluded from the analyses
Reproducibility	All attempts to repeat the experiment were successful. All data analysis is described in detail in the Methods section of the manuscript.

Randomization	The samples were allocated into groups randomly.
Blinding	Studies were not blinded during data collection or analysis since we know the treatment of each group before data collection.

Did the study involve field work? ☐ Yes ☒ No

Reporting for specific materials, systems and methods

We require information from authors about some types of materials, experimental systems and methods used in many studies. Here, indicate whether each material, system or method listed is relevant to your study. If you are not sure if a list item applies to your research, read the appropriate section before selecting a response.

Materials & experimental systems

n/a	Involved in the study
☒	☐ Antibodies
☒	☐ Eukaryotic cell lines
☒	☐ Palaeontology and archaeology
☐	☒ Animals and other organisms
☒	☐ Clinical data
☒	☐ Dual use research of concern

Methods

n/a	Involved in the study
☒	☐ ChIP-seq
☒	☐ Flow cytometry
☒	☐ MRI-based neuroimaging

Animals and other research organisms

Policy information about studies involving animals; ARRIVE guidelines recommended for reporting animal research, and Sex and Gender in Research

Laboratory animals	The study did not involve laboratory animals, but the bacterial strains in this study are isolated from the phyllosphere of naturally growing alfalfa.
Wild animals	The study did not involve wild animals.
Reporting on sex	The study did not involve sex.
Field-collected samples	Bacterial strains attached to the phyllosphere of alfalfa in this study were transported back to the laboratory with ice, and separate at 4 ℃ in the laboratory and cultivate at 30 ℃.
Ethics oversight	No ethical approval or guidance was required, as the study did not involve animal policy.

Note that full information on the approval of the study protocol must also be provided in the manuscript.
